# Exploring User Visions for Modeling mHealth Apps Toward Supporting Patient-Parent-Clinician Collaboration and Shared Decision-making When Treating Adolescent Knee Pain in General Practice: Workshop Study

**DOI:** 10.2196/44462

**Published:** 2023-04-28

**Authors:** Simon Kristoffer Johansen, Anne Marie Kanstrup, Kian Haseli, Visti Hildebrandt Stenmo, Janus Laust Thomsen, Michael Skovdal Rathleff

**Affiliations:** 1 Center for General Practice (CAM-AAU), Department of Clinical Medicine Aalborg University Aalborg East Denmark; 2 The Rectorate at Aalborg University Aalborg University Aalborg Denmark; 3 Department of Health Science and Technology, Faculty of Medicine Aalborg University Aalborg Denmark

**Keywords:** mobile health, mHealth, design, patient physician relationship, collaborative care, shared decision-making, adolescents, parents, knee pain, patellofemoral pain, Osgood Schlatter, musculoskeletal, general practice, primary care, mobile phone

## Abstract

**Background:**

Long-standing knee pain is one of the most common reasons for adolescents (aged 10-19 years) to consult general practice. Generally, 1 in 2 adolescents will continue to experience pain after 2 years, but exercises and self-management education can improve the prognosis. However, adherence to exercises and self-management education interventions remains poor. Mobile health (mHealth) apps have the potential for supporting adolescents’ self-management, enhancing treatment adherence, and fostering patient-centered approaches. However, it remains unclear how mHealth apps should be designed to act as tools for supporting individual and collaborative management of adolescents’ knee pain in a general practice setting.

**Objective:**

The aim of the study was to extract design principles for designing mHealth core features, which were both sufficiently robust to support adolescents’ everyday management of their knee pain and sufficiently flexible to act as enablers for enhancing patient-parent collaboration and shared decision-making.

**Methods:**

Overall, 3 future workshops were conducted with young adults with chronic knee pain since adolescence, parents, and general practitioners (GPs). Each workshop followed similar procedures, using case vignettes and design cards to stimulate discussions, shared construction of knowledge and elicit visions for mHealth designs. Young adults and parents were recruited via social media posts targeting individuals in Northern Jutland. GPs were recruited via email and cold calling. Data were transcribed and analyzed thematically using NVivo (QSR International) coding software. Extracted themes were synthesized in a matrix to map tensions in the collaborative space and inform a conceptual model for designing mHealth core-features to support individual and collaborative management of knee pain.

**Results:**

Overall, 38% (9/24) young adults with chronic knee pain since adolescence, 25% (6/24) parents, and 38% (9/24) GPs participated in the workshops. Data analysis revealed how adolescents, parents, and clinicians took on different roles within the collaborative space, with different tasks, challenges, and information needs. In total, 5 themes were identified: *adolescents as explorers of pain and social rules*; *parents as supporters, advocates and enforcers of boundaries*; and *GPs as guides, gatekeepers, and navigators or systemic constraints* described participants’ roles; *collaborative barriers and tensions* referred to the contextual elements; and *visions for an mHealth app* identified beneficial core features. The synthesis informed a conceptual model, outlining 3 principles for consolidating mHealth core features as enablers for supporting role negotiation, limiting collaborative tensions, and facilitating shared decision-making.

**Conclusions:**

An mHealth app for treating adolescents with knee pain should be designed to accommodate multiple users, enable them to shift between individual management decision-making, take charge, and engage in role negotiation to inform shared decision-making. We identified 3 silver-bullet principles for consolidating mHealth core features as enablers for negotiation by supporting patient-GP collaboration, supporting transitions, and cultivating the parent-GP alliance.

## Introduction

### Background

Approximately one-third of adolescents (aged 10-19 years) experience long-standing musculoskeletal pain [1,2]. At the age of 10 years, there is a sharp increase in adolescents consulting their general practitioner (GP) owing to a musculoskeletal complaint [3]. Between the age of 12 and 15 years, musculoskeletal complaints are the fourth most common cause for consulting general practice [4]. The most common pain site is the knee, accounting for between 30% and 50% of all presentations in this age group [5]. Previously, knee pain was considered to be benign and self-limiting [6], but recent studies have demonstrated that 40% to 50% of adolescents still struggle with pain after 2 to 5 years [7], and 7 out of 10 had reduced or halted their sports participation owing to knee pain [8]. This is problematic because life course studies highlight adolescence as a transition period [9], where health habits are formed and carried into adulthood [10]. Interventions with exercises and leaflets with patient education on managing knee pain have shown potential for improving adolescent’s prognosis [11-14], but maintaining adolescents’ performance with exercise and self-management activities remains as a barrier to success in this patient group [11]. Ensuring that adolescents learn to effectively self-manage their knee pain is important to enable patients to gain corrective experiences while reducing the period with experienced limitation [15,16].

### Mobile Health Apps

Mobile health (mHealth) apps are promising tools for improving the treatment of adolescents with everyday management needs owing to chronic conditions [17,18], and their acceptability of the technology is considered to be high owing to their common use of mobile phones [19]. Defined as “health practices supported by mobile and wireless devices” [20], mHealth apps draw upon the always-present, always-on properties of smartphones [21] to deliver just-in-time health interventions, text reminders, tailored information, self-tracking, connectivity, and decision-making support to contexts where patients experience their conditions [22]. Although literature highlights how the inclusion of mHealth may foster more patient-centered treatments [23], the evidence for mHealth’s efficacy in creating positive health outcomes and behavior change in patients remains indicative and contradictory [24]. Systematic reviews (2013-2020) have documented how including mHealth apps in the treatment of adolescents were associated with improvements in disease awareness, self-management abilities, treatment adherence, psychological well-being, and behavior change across conditions (asthma, diabetes, arthritis, and psychological issues), but the findings were inconsistent [25-33]. Qualitative studies support that mHealth can assist the development of personal management strategies and assist young patients in engaging with clinicians in cocare situations [19,34,35]. However, very few provide guidance on how future mHealth apps should be modeled to be integrated into complex treatment settings as tools for enhancing existing treatments and facilitating continual care [36-38].

### Self-management and Shared Decision-making

Self-management is essential for achieving recovery from knee pain [39]. Clinician-delivered patient education has been hailed as effective for teaching patients to self-manage their knee pain [13,40], but health literacy studies point to adolescents’ here-and-now perspective on injuries, their desire for independence, and capacity for understanding GPs’ instructions as barriers when supporting young patients [41,42]. Involved parents can help adolescents’ transition to self-management, through task assistance, coaching, guidance, rewards, and help during management mistakes [43-46], but this requires agreement on tasks and responsibilities to enable collaboration [43-47]. Shared decision-making holds the power to engage multiple stakeholders in the planning and facilitation of care, by merging patient and caregiver preferences with evidence-based practices [48], and the concept is central to the collaborative care process [49]. Exploring the visions of adolescents with long-standing knee pain, parents, and GPs about how an mHealth app can support individual and collaborative self-management when adolescents receive GP treatment for their knee pain may identify targets for designing mHealth tools, which are easy to be integrated into existing treatment practices, and empower identification and resolution of management and adherence barriers through shared decision-making [37].

### Objectives

This study aimed to identify principles for designing mHealth core features, which are sufficiently robust to support adolescents’ everyday management of their knee pain and sufficiently flexible to act as enablers for supporting patient-parent-GP collaboration and shared health decision-making.

## Methods

### Study Design

Action research was included as a methodological framework to guide our application of methods, analysis, and knowledge production [50,51]. The project’s intervention component consisted of 3 future workshops [52,53]: 1 with young adults with knee pain since adolescence, 1 with parents of adolescents with knee pain, and 1 with general practice physicians. Participants’ dialogues were captured via audio recorders and analyzed separately using reflective thematic text analysis [54] to map the general challenges and visions of each participant group for an mHealth app. The extracted insights and visions were synthesized in a matrix, to identify lanes of collaboration and tension sources and to facilitate the crystallization of design principles [55]. The study was reported in accordance with the guidelines in the CONSORT-EHEALTH (Consolidated Standards of Reporting Trials of Electronic and Mobile Health Applications and Online Telehealth) checklist, to ensure that our communication of findings corresponded to domain specific standards (Multimedia Appendix 1 [1-4,6-9,13,19,34-61]).

### Ethical Considerations

The study protocol was submitted for revisions to the regional board of research ethics in Northern Jutland, and they ruled that the project was permitted to continue without registration based on national guidelines.

### Participants

We included 3 study populations that were separated in terms of their roles in the clinical setting—patients, clinicians, or next of kin. Young adults (aged 18-25 years) with long-standing recurring knee pain during adolescence (emerging age 10-15 years; duration >6 months) were included into study population 1. The decision to include young adults was rooted in how self-management skills are developed over time [56] and how young adults would be capable to critically reflect on how mHealth features could have supported their transition to self-management. Exclusion criteria included competing musculoskeletal or pain conditions unrelated to knee pain, long-term illness lasting >3 months, psychological issues that required medicine and surgery of the knee. Parents of adolescents with knee pain (emerging age 10-15 years) were included into study population 2. We decided not to include parents of participants in population 1, as these participants were legal adults and we deemed that the challenges related to supporting adult children were outside our scope. Exclusion criteria included competing musculoskeletal or pain conditions, severe physical handicaps, psychological issues, and surgery of the knee. Finally, GPs were included into study population 3. Inclusion criteria were employment in general practice for at least 1 year, experience in treating adolescent knee pain, and willingness to participate. Participants for study populations 1 and 2 were recruited through social media posts targeting individuals in Northern Jutland, containing the link to a form with questions related to the inclusion criteria, contact information, and consent forms. Potential participants, who expressed interest in participating and consented to contact, were contacted via phone by SKJ; screened; provided informed about the project, participants’ rights, and data treatment procedures (oral and written); and recruited. Participants in study population 3 were identified within the Center for General Practice and Nord-KAP—the Quality Unit for General Practice in Northern Jutland’s clinician networks, contacted via email and phone, informed, screened, and recruited into the project.

### Future Workshops

We drew upon the future workshop as described by Jungk and Mullerts [62] as a template for planning our study’s intervention component. A key feature of the future workshop relates to the use of coconstruction of knowledge, through collaborative activities and ideation to extract novel and useful solutions to complex real-world problems. Future workshops use a 3-step process, entailing critique, fantasy, and defining shared visions [52] to guide participants’ dialogues toward formulating shared visions for possible futures (refer to Table 1).

To facilitate this transition between the workshop phases, a generative activity was designed, which used case vignettes [63] and inspiration cards [64] to encourage participants’ dialogues and guide them through to the third and final phase of our future workshops. Furthermore, it was decided to forgo the final phase (follow-up phase), because implementation was outside our scope of inquiry.

**Table 1:** Overview of the future workshop phases^a^.

Timeline and phases			Brief explanation about the phases
**Before**			
	Preparation	Organizers and facilitators agree on the theme, invited participants, methods, location, locales, rules, and timetables of the future workshop.	
**During**			
	Critique	Participants investigate the problem through criticism and brainstorming. Challenges and ideas are noted and organized into themes.	
	Fantasy	Participants create a picture of a utopic future. Brainstorming and creative techniques are included to suspend criticism and extract ideas.	
	Implementation	Participants organize, evaluate, and develop ideas related to practicality and ease of implementation. Action plans are developed.	
**After**			
	Follow-up^b^	Action plans are monitored, changes are performed, and new future workshops are planned to address challenges to implementation.	

^a^Phases and descriptions are adapted from a paper by Vidal et al [53].

^b^The follow-up phase is not included in this paper.

### Case Vignettes

A case vignette (Multimedia Appendix 2) was designed in collaboration with young adults with knee pain, parents, and GPs to outline salient features of young patients, seeking treatment for their knee pain in general practice. The case was included to initiate discussions, by presenting a patient narrative with relevant and irrelevant information [63]. The case was tested and iterated with 3 GPs, 2 parents of adolescents with knee pain, and 2 young adults with knee pain to ensure comprehension.

### Inspiration Cards

An inspiration card exercise was developed to encourage dialogue and cocreation and guide participants through the future workshops’ 3 phases (Multimedia Appendices 3 and 4). The inspiration card game featured themes related to 3 conceptual domains: physical domains, problems or challenges, and possible solutions. The themes were identified by 3 members of the project group (SKJ, MSR, and JLT) and reflected our initial understanding of the challenges and experiences encountered by members of all 3 study populations. The inspiration cards were tested and iterated following the same pattern as the case vignettes to ensure relevance of themes and comprehensibility.

### Other Artifacts

Participants in each group were provided with other artifacts such as post-it notes in 3 colors (red, yellow, and green), pens, and pen markers, which participants could use to brainstorm ideas; organize emerging themes to visualize conceptual relations; and engage in a shared evaluation of themes, concepts, and novel ideas. To support participants in bridging the gap between ideation and visioning (future workshop phases 2 and 3), the facilitators advised groups to rearrange, explore, and prioritize their ideas by reorganizing the design cards before engaging in the work of phase 3.

### Setting and Procedure

Special care was taken to ensure that all 3 workshops followed the same procedure to heighten the compatibility of the extracted insights and visions. Workshops 1 and 2 (young adults and parents) were conducted at a local community center, whereas workshop 3 was conducted at the Center for General Practice in Aalborg. All workshops lasted approximately 3 hours, distributed across three 40-minute phases and brakes. Each workshop was conducted with a primary coordinator (SKJ), a workshop facilitator who introduced workshop activities and guided participants through the 3 phases, and 2 cofacilitators (AMK, MSR, and JLT) who would help the facilitator in guiding group discussions and otherwise observe the process from the background. Upon arrival, participants were divided into work groups of 3 to 4 participants each. Each workshop was initiated with a short introduction by the facilitator and a presentation by an invited specialist, physiotherapist, mHealth specialist, and eHealth specialist. The facilitator would then introduce the case vignettes and the inspiration cards corresponding to the given phase and provide instructions about how to complete the exercises of each phase. This procedure was repeated before each of the 3 phases (critique, ideation, and vision phases) of the workshops. Each phase was concluded with a plenary discussion, during which the groups presented their thoughts and ideas, while the facilitator summarized key points on a flipboard and asked follow-up questions. Upon completion of the final phase, all groups presented their visions for an mHealth app for feedback from other participants and facilitators. The workshops concluded with a debriefing session, during which the participants were informed about their rights, completed the consent forms, and were given the opportunity to ask final questions.

### Data Collection

Overall, 3 types of data were collected to illuminate the problem from different angles. Clinical characteristics of study populations 1 and 2 were collected using web-based forms, whereas core data from population 3 were collected through phone interviews. During workshops, participants’ visions and insights emerging from plenary discussions were noted on flipboards by the facilitator, and group discussions were captured via audio recorders for analysis and interpretation using reflexive thematic analysis (RTA).

### Analysis

The data gathered during the 3 future workshops were analyzed through RTA by Braun and Clarke [54] by the lead researcher (SKJ) and 2 student workers (KH and VHS). The data sets from each individual workshop were transcribed for meaning retention, as described by Kvale and Brinkmann [65], using Expresscribe (NHC Software) transcription software. The transcribed data sets were analyzed in parallel through a 4-stage process including familiarization, coding and identification of themes, condensation and refinement, and synthesis into a shared narrative (Multimedia Appendix 5). NVivo (version 11; QSR International) coding software was used for the coding and organization of themes, and coding lists were created and maintained by all coders (SKJ, KH, and VHS) during each individual analysis. Identified themes were refined through iterative cycles of horizontal readings and condensation, and related subthemes were merged. Emerging thematic overlaps, divergencies, and relationships identified within the individual analysis were discussed among the lead researcher (SKJ) and student workers (KH and VHS) until consensus was reached. Considering the thematic relationships identified during each individual analysis (Multimedia Appendix 6), a narrative of 5 storybook themes was identified. Consecutively, the insights uncovered during each thematic analysis were organized in a matrix to map collaborative tensions, identify design principles, and inform a conceptual model [55]. To ensure coding integrity, stakeholder checks were conducted by the lead researcher (SKJ) and student workers (KH and VHS), and coding list entries were discussed as the analysis progressed. The lead researcher (SKJ) was responsible for the final abstraction and presentation of the findings in the 5 storybook themes, which outlined the narrative in the *Results* section. All involved parties (SKJ, KH, and VHS) approved the storybook themes, narrative, matrix analysis, and conceptual model before the analysis was concluded.

## Results

### Inclusion of Participants

**Figure 1:**
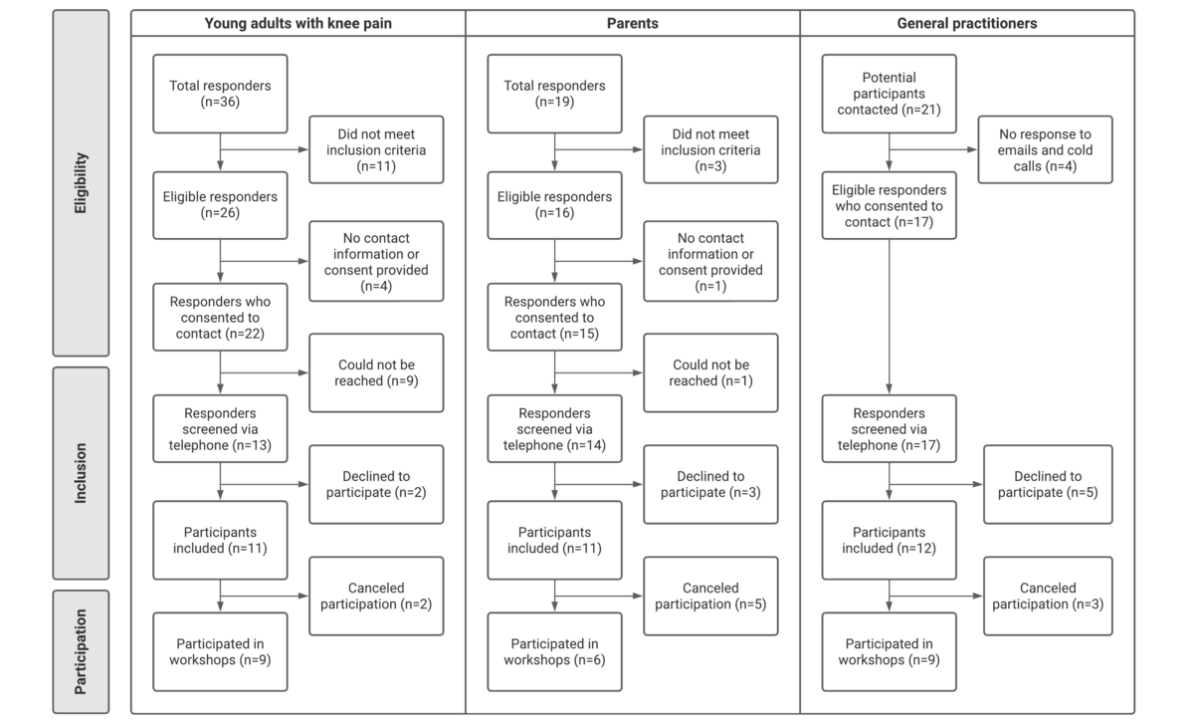
A flowchart providing an overview of the 3 lines of inclusion from when participants responded to our outreach efforts (social media posts or emails).

The social media posts and phone screening generated 36 potential participants for workshop 1 (young adults) and 19 potential participants for workshop 2 (parents). Our efforts to contact GPs in the Northern Jutland area via emails and cold calling yielded 17 potential participants from the 21 who were initially contacted (Figure 1). From the 11 included young adults, 9 (82%) participated (n=8, 89% women; mean age 20, SD 1.73; range 18-23 years), whereas 2 (18%) withdrew their participation. All participants in workshop 1 (9/9, 100%) had experienced long-standing knee pain, emerging between the age of 11 to 16 (mean 13, SD 1.32) years and lasting for an average of 5.8 (SD 2.45; range 3-9) years. From the 11 parents included, 6 (55%) participated (n=5, 83% women; mean age 46, SD 1.44; range 41-52 years). Parents reported how all their adolescents (mean age 11, SD 1; range 10-12 years) had experienced knee pain for an average of 2 (range 1-6) years and how 67% (4/6) of participants had consulted their GP, 33% (2/6) had consulted physiotherapists, and 33% (2/6) of the parents had not yet sought treatment for their child’s knee pain. From the 12 included GP’s, 9 (75%) participated (n=4, 44% women; mean age 42, SD 11.84; range 30-63 years), whereas 3 (25%) canceled in advance. The participants in workshop 3 had an average of 8.5 (SD 7.8; range 1.5-25) years of experience in general practice, with 58% (7/12) of them reporting having a special interest in musculoskeletal conditions.

### Results of the Data Analysis

#### Overview of Themes

RTA uncovered a narrative of 5 storybook themes. Overall 3 themes described the roles participants played within the treatment situation, and 1 theme described the collaborative barriers and challenges across contextual settings. Theme 5 identified core features and collaborations based on the participants’ visions for an mHealth app. The insights from the analysis were summarized within a matrix to inform a conceptual model, identify principles for expanding the design of mHealth core features, and enable patient-parent-GP collaboration and shared decision-making.

#### Theme 1—Adolescents as Explorers of Pain and Social Rules

The first theme comprised statements describing how participants experienced their emerging knee pain and the challenges related to the everyday management of knee pain. The analysis revealed how young adults described their emerging knee pain as fluctuating or something that emerged in different situations such as stair climbing, bicycling, running, sports, and gym class and affected the adolescent’s ability to engage in valued activities. The young adults described being tasked with exploring ways to cope with emerging pain and pain-related frustration, while managing the social consequences of being limited. A common theme during workshop 1 was how emerging knee pain initiated a *vicious cycle* in which adolescents’ efforts toward minimizing the social consequences or hiding their condition resulted in increased pain. A young adult described the cycle as follows:

There is this vicious circle, where you start feeling the pain during sports, talk about it at home, go to the doctor, and then the doctor tells you that there is nothing. You go back to everyday life again, return to school, try to spend time with your friends and start to feel pain again. You withdraw for a little while, and start being left out of your [friend] group. So, you return to sports to get back into the group and the cycle continues.

Participant 3

One of the main challenges described, related to the invisibility of knee pain and how the adolescents were dependent on others recognizing their pain (peers, parents, and coaches) to avoid being branded as lazy, whiney, or careless. Another recurring theme related to how fear of being “benched” may result in adolescents “forgetting” or ignoring their pain to fulfill social obligations or avoid exclusion. A participant described how she considered ignoring her pain to avoid missing out of activities with friends:

Well, it is possible that you might forget to tell others about your [knee] pain, because you’re afraid that they’ll think you’re a cry-baby or that you won’t be allowed to participate in things you’re normally allowed to...We were in Africa one winter, and the team went to climb a mountain and I had to wait by the foot. Back then I considered not telling [the others] that I had pain, so I could come along.

Participant 5

Alternatively, 1 group (workshop 1) described how acceptance from others or honesty about the knee pain was important and empowered adolescents to stop hiding the pain and focus on managing the condition. A participant articulated the link between gaining parents and GP’s acceptance and managing the knee pain in other situations:

It’s more like a step on the journey towards gaining this acceptance from the world, but when you have the backings of your parents and the doctor...I think that makes it easier to manage it [knee pain]. I definitely remember, how it was easier to manage [the knee pain], when my mother was involved.

Participant 2

Apart from acceptance, participants described performing regular knee exercises and learning to “find the limit” with their knee pain as essential for breaking the vicious cycle and balancing self-care while performing everyday activities and how this was challenging for adolescents. Although young adults and parents highlighted adolescents forgetting their knee pain in nonimpact situations or losing faith in exercises as barriers to breaking the negative spiral, young adults and GPs emphasized how learning to differentiate between good and bad pain is essential for managing the knee pain:

I was always told that I just needed to be warmed up, so I ended up thinking doing sports was equal to having knee pain, and therefore I never really learned to find the limit where I should have stopped in relation to the pain I felt. The result...I would come home from training and have to lie down with my leg up because I was in pain.

Participant 9

Finally, young adults, GPs, and parents highlighted how adolescents may struggle to remember and expressing their pain in words when asked by parents or GPs. This posed a challenge when reaching out to parents or health care professionals for support in managing their knee pain. One group of participants (workshop 1) highlighted fear of stigma as a contributor to this problem, whereas another group’s (workshop 1) comments indicated that adolescents lacked the vocabulary for describing their pain beyond the immediate pain experience. A participant (workshop 1) described it in following way:

I also found it difficult when my physio would ask the question; where do you get pain, what is it that cause you pain, and what does it feel like?...I don’t know, because in this moment I don’t have any pain. So I can’t give you an explanation on how the pain is...

Participant 5

#### Theme 2—Parents as Supporters, Advocates, and Enforcers of Boundaries

A recurring theme during the second workshop was parents referring to taking on the *parent role* to solve a problem. Although the parents generally recognized the adolescents as individuals with their own opinions, experiences, and desires, the *parent role* term was often used in recognition of how certain aspects of management were difficult for adolescents. Thus, parents had to step in and take control to remove barriers that inhibited adolescents’ management of their knee pain. A parent described the *parent role* in terms of supporting their child:

My role as a parent is to take her [daughter] seriously...To do the right thing a hundred precent...this includes seeking out everything [treatments] to find out exactly what this [the knee pain] is. To back her up 100% precent, all the way through the health system.

Participant 14

Another participant exemplified how taking the parent role also meant stepping in and setting boundaries when they felt their adolescents were not able to do so themselves. A parent described setting limits for her son’s participation in soccer:

Sure, I could tell my son that [he had to take brakes], but I’m sure he would just lie his way out of it. Well, he can’t do that right now, because I often accompany him during training and matches...You’re not match ready, since I am the one deciding this...Still, it’s hard to keep them away from it [sports] because this is what they are really keen on doing.

Participant 10

Although parents from both groups highlighted being present, listening, taking complaints seriously, and setting boundaries as important for supporting their adolescent’s health decisions, both groups described alternating among the 3 tasks of emphasizing with the adolescents, advocating for the adolescents, or reassessing their own understanding of knee pain to support their adolescents by creating situations where adolescents were capable of self-management. Furthermore, parents described having experienced how their adolescents struggled to remember, understand, and express their knee pain in words. This complicated the parents’ task of assessing when to seek treatment, resulting in parents overlooking or negating adolescents’ attempts to express their knee pain. Thus, the parents had to learn to read between the lines, within the adolescents’ descriptions. This need was exemplified in a parent’s description of discussing the knee pain with her daughter:

I always had to ask my daughter how bad is it? She doesn’t really complain about it [knee pain] except for what she tells me when we were in these situations...and then she’ll just tell me: but mom, I just think I’ve gotten used to it [knee pain].

Participant 11

In terms of advocacy, parents in both groups described instances where they had stepped in and negotiated on their adolescents’ behalf and how this advocacy initially occurred in the clinical setting and extended into the parents and adolescents’ networks after consultations. Parents described that negotiation with the GP aimed at supporting adolescents in articulating their pain and ensuring that their child benefited from their consultation. Parents described how advocacy also included withdrawing from treatment or seeking alternative treatments and information sources if they felt invalidated or that the GP did not meet their needs during consultations. A parent described how her expectations had prevented her from seeking additional treatments for her daughter:

During spring we had a longer period where I thought that we might have to take her to a GP [for the knee pain], but where our own GP who we have been seeing for years ended up quitting...and I just thought why bother because then would have to see a new one. I know the old GP would have taken it into account if I told him that we had waited and seen for a long time. We had waited for three months...But it was not him anymore so I thought we wouldn’t bother.

Participant 10

#### Theme 3—GPs as Guides, Gatekeepers, and Navigators of Systemic Constraints

The data from workshop 3 revealed several tasks, responsibilities, and dilemmas, which GPs had to navigate when treating adolescent knee pain. GPs described taking on the role as teachers or coaches, tasked with guiding the adolescents into a positive spiral with decreased somatization; better disease management; and sustained, balanced participation in sports as their main goals when treating youths with knee pain. Through this, the GPs have to balance the tasks of managing the adolescents’ pain in situ, setting a stage for self-management in the future, gatekeeping, and navigating systemic constraints. However, the GP’s main goal was described as ensuring that adolescents learned to manage their condition, as described in the following quote:

What are we trying to archive? It is, that the patient [adolescent] becomes better at managing his situation. To do this, patients could benefit from becoming more knowledgeable and like being able to say; Hey...it also hurts when I’m not exercising and I believe there is a learning in this.

Participant 22

The participants’ statements during workshop 3 indicated how treating adolescents with knee pain was a 2-step process and how ruling out serious pathologies or trauma, diagnosing the condition, identifying the right treatments, informing, and managing expectations was part of the initial step of treatment. A GP described how identifying alarm symptoms was important:

Yes, we need to know the alarm symptoms...Are there any symptoms we professionally know that; “Oh this, this we need to effectuate on immediately if we spot it.” We need some kind of screening feature for what is acute, what is dangerous and not. We are doctors, that’s why patients come to us in the first place.

Participant 16

Besides momentary management, the analysis revealed how GPs developed and used different behavioral strategies in tandem with usual care, to encourage the adolescents to explore, gain insights, and gradually become better at making health decisions going forward. A GP described the strategies he used to supportive strategies:

Something that could be really beneficial is to explain to people how the pain emerges...I sometimes use the term “Pain memory,” that you can have pain on an injury that is almost fully healed, but you will continue to experience pain right? So sometimes it can be useful to show them that it [their knees] cant break. Some people have a belief that things may like fall apart.

Participant 17

Although one GP group highlighted how this required understanding the “full patient,” other strategies included addressing the patients’ worries and concerns to facilitate acceptance, encouraging trial and error by providing suggestions for managing pain fluctuations, exercising, motivating adherence via goal setting, and establishing working alliances with parents. However, GPs highlighted that their efforts toward supporting future self-management depended on whether adolescents felt that following the GP’s advice allowed them to better understand and control their pain. A GP described the importance of understanding the whole patient with the following quote:

You need to look at the “whole patient” like her [the case]...what she wants to archive. I normally differentiate between the lazy bodies and the non-lazy ones. With the lazy bodies, the problem is often that they will state that they have pain, because they might stand to gain from it...like I can’t participate in gym class or bicycle to school.

Participant 17

GPs identified several constraints within the treatment situation that had a direct influence on the GP’s treatment decisions and possible outcomes. The long disease course of knee pain and patients slipping through the cracks were highlighted as the main concerns and challenges of the GPs. Moreover, GPs pointed to adolescents’ difficulties in articulating their knee pain and patients or parents’ misunderstanding of GP’s instructions as barriers, which contributed to adverse outcomes such as dissatisfaction, withdrawal from treatments, and parents insisting for surgery. A GP described how knowing when his message got across to the adolescents was a challenge. Other GPs suggested that forming a therapeutic alliance with parents could help facilitate the knowledge translation, avoid withdrawal, and provide GPs with the ability to monitor and adjust their treatments by proxy. A GP elaborated further on this, by stating how maintaining adherence was ultimately the patients and parents’ responsibility:

But the problem is all too real in the clinic, as a lot of things will disappear within a short time, but that again means that we should be better to provide patients a safety net if it [knee pain] continues. But they also have a responsibility for coming back again [if pain persists]. I can’t take on that responsibility, all the time.

Participant 19

#### Theme 4—Collaborative Barriers and Tensions

Our workshops uncovered how adolescents, parents, and clinicians engaged in different types of collaboration aimed at empowering adolescents to enter an upward spiral with increased understanding of the disease and self-management. The analysis identified several communicative barriers, which lead to tensions in parent-patient-GP communication. Young adults, parents, and GPs highlighted adolescents’ difficulties in remembering and verbalizing their knee pain as a major source of tension. GPs described how this acted as a barrier to fulfilling their role in terms of diagnosis, management education, and planning future treatments, and parents and young adults corroborated this, with young adults suggesting that adolescents’ inability to explain their knee pain may be related to lack of in-depth understanding of their knee pain. A GP suggested how pain diaries could be used to alleviate tensions, by helping adolescents to articulate developments in their pain:

Because, most 10- to 15-year-old adolescents, when you ask them to recall; How many or how often do you experience knee pain, which time of the week or whatever this might be, will have a hard time providing an ample description of this, so this way you may get an overview of how they are impacted by their knee pain...And maybe it could be combined with something [a feature] which gives an indication of their pain severity.

Participant 18

Another tension source was related to adolescents or parents’ expectations of obtaining a solution to the knee pain when entering a treatment collaboration. This was corroborated by young adults and parents, who described how being told “wait and see” could lead patients and parents to conclude that the GP did not believe them or know how to treat their knee pain. A parent described feeling invalidated after receiving the “wait and see” recommendation for her adolescent, and this had affected her expectations of future consultations:

Now the two of us are here where we haven’t quite made it to the GP’s yet...and we have discussed it and believe it boils down to us feeling that we weren’t heard when it...and being sent home and told to “wait and see.”...so the thought of us being sent back home again...well then, we might as well wait and see [by] ourselves.

Participant 10

GPs corroborated this during workshop 3, by highlighting how they knew there were limits to what they could do for adolescents consulting with knee pain and how managing parents’ expectations was quintessential when gatekeeping, to avoid parents becoming frustrated and seeking other treatments prematurely. GPs also discussed using imagery to give parents something tangible, build alliances, avoid withdrawal, and prevent parents from insisting for surgery:

It depends on how the parents are involved in this...If you can’t get an alliance with them before you have made a scanning and they are just like a white wall...like they’re simply not listening, and you know they’ll eventually walk out the door and seek out a private clinic or something, then I might open up the possibility of getting a scanning, but I generally believe that it [scanning] may potentially do more harm then good, because you might find something [unrelated].

Participant 18

Furthermore, GPs highlighted systemic constraints such as consultation times, subpar IT systems, and loss of communication owing to referrals as barriers leading to loss of contact with patients and parents:

We discussed how the condition may persist for a long time, potentially without a whole lot of doctor-patient contact...So when we are first made aware of the injury until they return...it could be months, even years apart before we see the patient again. And we haven’t had a chance to affect the outcome, apart from a few weeks’ time.

Participant 20

Parents described how their lack of knowledge about knee pain caused tensions when assessing whether additional treatment was merited, when advocating with the GP, and when communicating their adolescents’ conditions and forming alliances with actors in their networks (teachers, coaches, and other parents). Thus, parents and young adults described how parents’ lack of knowledge meant that they risked overlooking or negating adolescents’ symptoms, unnecessarily restricting their sports participation, or accepting nonbeneficial treatments. A parent suggested how tailored patient information could help parents to know when to seek additional treatments:

I would have liked having a guide for how long it takes...I know sundhed.dk [Danish government health portal] has something where you can describe your symptoms and whatever, and in the bottom I know they have something like...now its lasted for so and so long, and then should do this and this. If it looks like this, you need to contact your GP...like a guide of some sorts.

Participant 12

Parents and young adults described an emerging dynamic, which eventually led to the formation of an alliance in which parents helped adolescents to create space for their self-management in everyday situations. Although young adults described how parental recognition made it easy to confront teachers, peers, and coaches about knee pain, reaching out to parents meant risking being dismissed or restricted from sports participation, which created tensions. In contrast, ensuring their child’s well-being was highlighted as quintessential to parents, but their lack of understanding about knee pain sometimes led them to take the wrong actions when their adolescents presented pain. Parents described how taking a more trusting approach reduced tensions and allowed them to focus on gatekeeping; advocacy during GP visits; and engaging with teachers, coaches, and other parents to create space for their adolescent’s self-management. A parent articulated this in the following way:

But it comes back to what responsibility you have as a parent. Because you make the decision to enter actively into it [supporting the adolescent] and provide your input. And by this I don’t mean entering something into a dead system. You look the other person into the eyes and say; I have this issue with my child, can we work out a solution together.

Participant 14

#### Theme 5—Visions for an mHealth App

Finally, the analysis identified several visions for mHealth core features for enhancing collaboration and shared decision-making across collaborative spaces. The visions were distributed across 3 categories, directed toward enhancing reassurance, supporting pattern recognition and articulation of knee pain, and enhancing 2-way communication. However, participants described these core features as intersecting and needed to support different activities simultaneously for maximum effect. A GP described how his group envisioned that an mHealth app should support different tasks simultaneously:

I’m thinking that you could create a three-legged system. Like something with monitorization of, what’s the status of this [knee pain]. How is it developing. A tool for treatment as well as a patient education tool.

Participant 20

Participants envisioned an mHealth app containing features for reassuring adolescents and helping them to test and evaluate their management decisions, when the knee pain emerged in everyday situations. Both GPs and young adults suggested how a library (videos) with trustworthy information about knee pain mechanisms, possible trajectories, and a *first aid kit* for managing flareups could reassure adolescents, promote self-education, and allow them to share this knowledge with peers. However, the young adults suggested that patient cases with other adolescents with knee pain were more easily relatable for youths and could provide hope for betterment. Furthermore, GPs and young adults highlighted that adolescents sometimes struggled to remember and comply with exercise programs and that adding a library with exercise videos and in-depth explanations could reassure adolescents that they were performing the exercises correctly. Ensuring that exercises were actionable (short and easily understandable) while combining them with a tracking feature could motivate exercise adherence by visualizing the short-term and long-term effects of exercises:

Well, I did actually get started on some type of rehabilitation, but I eventually quit because I didn’t really feel that it worked...so if you’re thinking apps, then incorporating one [a feature] which provides you with suggestions for exercises and gives you reminders like “remember to make these exercises.”

Participant 9

Participants across all groups suggested having core features that empowered adolescents to monitor, explore, and identify patterns in their knee pain. Both young adults and GPs described how a journal feature could support adolescents’ self-management by helping them in identifying activities that caused pain. Nevertheless, young adults and parents suggested incorporating reminders and predefined pain scales to reduce the burden related to monitoring the knee pain. All participants suggested that visualizing journal entries could help adolescents in overcoming their challenges by remembering pain-causing activities and articulating their knee pain when it emerged. Young adults suggested how incorporating a map visualizing common developments in the knee pain could assist adolescents and parents in assessing how the knee pain progressed and establishing treatment goals. This was corroborated by GPs who described how this feature could help adolescents in identifying activities that would not affect their knee pain:

...And then there was something with a pain measurement [feature], where you could note it as logbook with where you had pain and how much pain you had, but a combination of them, where you could get the connection between...I have this pain, maybe it subsides when I’m not active.

Participant 1

Finally, participants envisioned how core features could be expanded to enable negotiation of meaning and shared decision-making, but this required a balance because actors had different information needs. Participants generally agreed that the journal and visualization features were central to this, by providing GPs and parents’ insights into the adolescent’s experience. Young adults and GPs described how visualizing journal entries could help resolve tensions in GPs and adolescents’ communications during clinical visits by providing a common ground for discussions, whereas GPs and parents described how visualizations could also help GPs in adjusting treatments to the patient’s needs. However, GPs described how this required visualizations to be aggregated for easy overview to avoid time loss. Another vision related to the exercise library was how incorporating a checklist with symptoms to look out for could help parents in deciding when to seek additional treatments and prepare parents for engaging with coaches, teachers, GPs, and physiotherapists. Finally, GPs and parents described how an mHealth app could facilitate information flow during transitions between treatments or when negotiating with external actors (physiotherapists, coaches, and teachers), by alleviating tensions related to parents or adolescents forgetting information obtained from clinicians between consultations. This was corroborated by the young adults, who exemplified how an app could facilitate an ongoing negotiation among multiple actors, to ensure acceptance of the knee pain. This was exemplified in the following quote:

We discussed how it [an app] should be a little bit like “school-parent communication software”...but in a way where you have communication between the patient and the GP, and where the GP can post your exercises along with comments, videos or whatever...This way we get that acceptance of how the pain is real, which means the surrounding world are in on accepting them, but you need to start with the ones who are closest, like mom and dad.

Participant 2

### Results of the Matrix Analysis

#### The Conceptual Model

**Figure 2:**
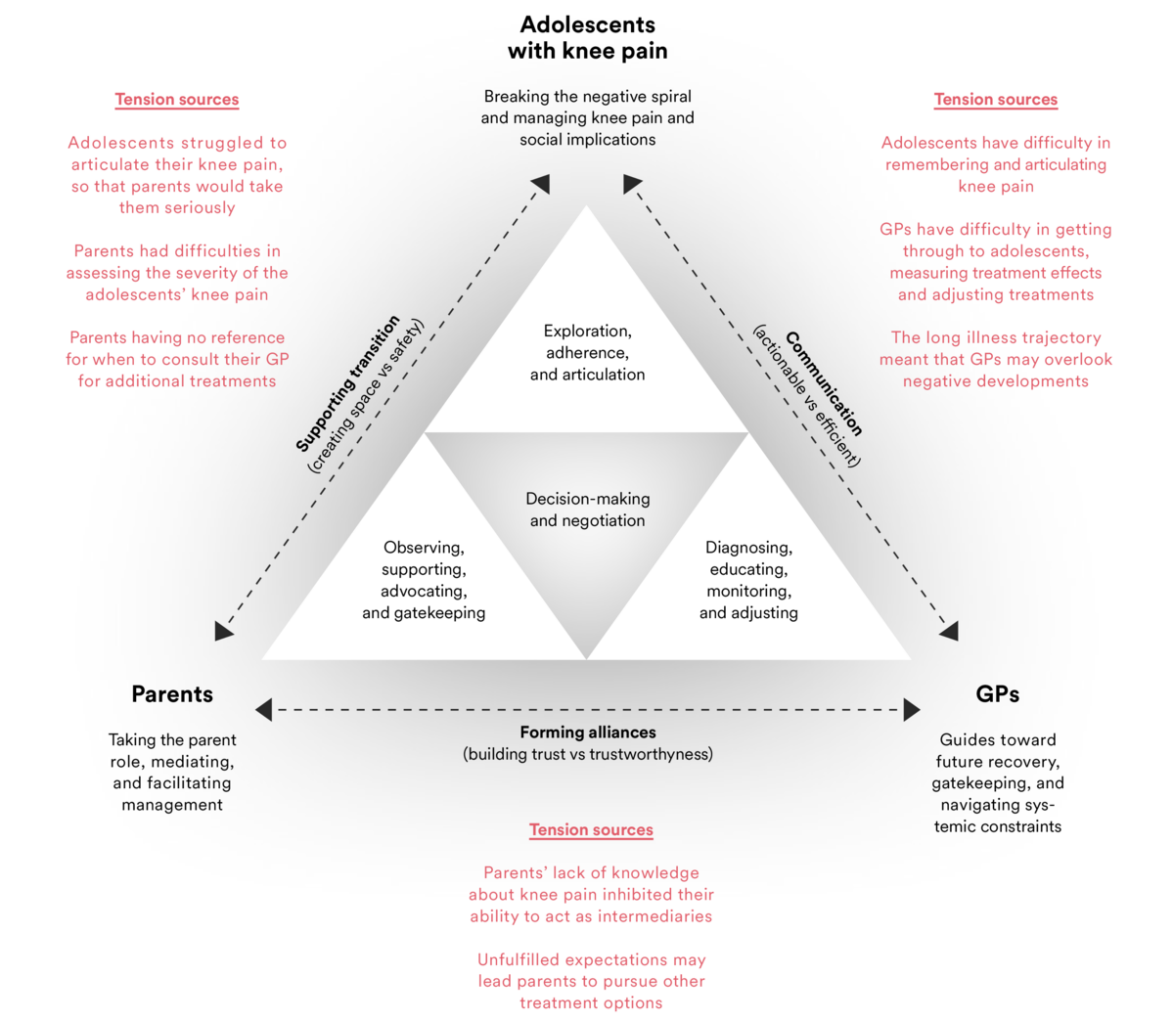
The conceptual model that was designed to illustrate the complex interplay between participants’ roles, their proximal and distal goals, management tasks, and barriers present in the collaborative space. GP: general practicioner.

The matrix analysis informed the construction of a conceptual model. Organizing participants’ descriptions about their roles, tasks, challenges, and interactions within a system identified how adolescents, parents, and GPs were interconnected within a triadic relationship, where all actors engaged in different modes of management behaviors (Figure 2). Considering the identified tension sources, the model outlined targets for designing mHealth core features to bridge the gap between the supporting participants’ individual management practices and collaboration across multiple contexts.

#### Decision-making and Negotiation

The layout with the embedded triangles illustrated that the participants’ collaboration in managing the adolescents’ knee pain unfolded at the individual and community levels across multiple contexts. A key insight was not only how participants took on different roles, tasks, and responsibilities within the collaborative space but also how these roles were often dual-sided and contradictory in nature. The individual triangles (top, left, and right) were designed to illustrate how the actors (adolescents, parents, and GPs) navigated these role-based contradictions via their management decision-making (center) in their individual contexts—an act that was obscured to other actors unless disclosed via words or observable actions. The matrix analysis identified how all actors encountered management barriers, which they could not resolve themselves (eg, obtaining a diagnosis, gaining knowledge about knee pain, and securing social support) and caused tensions in the collaborative space (Multimedia Appendix 7). To overcome these barriers, actors engaged with other actors to draw upon their competencies (as adolescents with knee pain, parents, and GPs) to expand their management capabilities (decision-making), modify contexts (eg, being excused from gym class and creating a working alliance), or adjust their roles and tasks in the collaborative space. However, participating in these exchanges meant *renegotiating* the participants’ individual goals, roles, and tasks to be effective (center and inner triangles). When successful, the negotiation may strengthen the actor’s individual health decision-making capabilities, articulate shared *goals*, and cultivate working alliances. Nevertheless, failure to negotiate was identified as having a cascading effect, leading to increased tensions and complexity in the collaborative space, inhibiting shared decision-making, and prompting treatment withdrawal if the tensions were not resolved.

#### mHealth Core Features as Collaborative Enablers

Considering participants’ visions for an mHealth app, described challenges, and identified tension sources (Multimedia Appendices 7 and 8), the matrix analysis identified several touchpoints where participants interreacted to resolve individual management challenges and how these interactions contributed differently toward sustaining the collaborative situation. This informed 3 principles for organizing mHealth core features as collaborative enablers for supporting tension reduction by empowering negotiation and informing shared decision-making.

#### Enhancing Communication

The young adults, parents, and GPs envisaged how health information collected via quantified self-tracking could support adolescent-GP communication and how their information needs differed in terms of timing, timelines, and modalities. Participants described how the act of self-tracking knee pain via, for example, pain journals, receiving tailored patient information (etiology and exercise support), and performance feedback, could help adolescents in assuming the role of explorers through the identification and articulation of patterns in their knee pain. However, this required that the delivered health information should be actionable in everyday settings to encourage exploration, compliance, and articulation. Furthermore, visualizations of aggregated self-tracked data could help adolescents and GPs to overcome communicative barriers by assisting adolescents in recalling and articulating previous developments in their knee pain, while simultaneously giving GPs a foundation for guiding the adolescents—by providing GPs an overview of the adolescents’ trajectory, the ability to monitor the effects of treatments and exercise regimes, and a starting point for discussing future treatments. However, effective presentation and delivery of the self-tracked health information were crucial to ensure GP use in complex clinical settings.

#### Facilitating Transition

Young adults highlighted how acceptance and adapting an honest perspective about knee pain was important for facilitating adolescents’ transition to self-management and how parental support could help adolescents to take on the role as explorers. The analysis revealed how different types of static information (patient cases, lists of symptoms, exercise videos, and patient information) could promote safety in making management decisions (individual level) by providing reassurance, along with vocabulary and expert information that adolescents could include when explaining their knee pain to peers, coaches, and GPs to avoid stigma. For parents, static information delivered with the app (eg, leaflets or webpages targeting parents and adults) could empower them to create space for adolescents’ exploration of their knee pain (decision-making) and remove management barriers through negotiations with other parents, teachers, coaches, and GPs. This included enhancing parents’ knowledge about knee pain symptoms and treatment types, while providing them with guidance and tools for how to engage and educate other actors, coaches, teachers, and other parents about knee pain and the management needs of their child.

#### Forming Alliances

Finally, the analysis outlined how communicative difficulties between GPs and parents could lead to tensions and parents deciding to withdraw and seeking other forms of treatments and how this was driven by parents not feeling seen or heard when consulting GPs. Both parents and GPs suggested incorporating core features that could help parents and GPs in entering negation and building alliances. Including a checklist for parents with symptoms and questions for GPs could limit tensions by ensuring that parents felt heard during consultations, while providing GPs space for addressing parental expectations to treatments. Furthermore, providing parents with information materials (folders and webpages) about the adolescent’s symptoms, treatment options, and prognosis could help them to adjust their expectations, while cultivating a sense of co-ownership and forming treatment alliances with the GP.

## Discussion

### Principal Findings

Our findings revealed several key insights that should be considered when designing mHealth apps as tools for facilitating patient-centered treatment of adolescents with knee pain in general practice. Our analysis indicated how adolescents, parents, and GPs entered a triadic relationship with different goals, tasks, and information needs, similar to what Hohmann [57] and Brooker [58] observed in pediatric settings. Participants worked toward 1 outcome—ensuring that the adolescent entered an upward spiral with decreasing pain and increasing control. Adolescents actively facilitated this transition at the individual level, through their exploration of their knee pain in the present [56,59]. In contrast, parents and GP’s roles were peripheral and focused on supporting adolescents in navigating future management obstacles through observation, encouragement, boundary setting, and provision of management advice and information. Our analysis showed how all actors alternated between 2 modes of management behaviors. This included making individual management decisions to overcome contextual management challenges and engaging with other actors to use their expertise (as adolescents, parents, and GPs) to adjust management practices or collaborations—an act that involved a renegotiating of goals, tasks, and responsibilities to be successful. Although negotiation acted as a linchpin for shared decision-making [60], our analysis identified how articulation, lack of knowledge, unfulfilled expectations, and nonreciprocity inhibited negotiation and increased tensions in the collaborative space. Participants envisioned how an mHealth app for adolescents with knee pain should focus on providing reassurance, pattern recognition, and facilitating 2-way communication. Our conceptual model identified 3 principles for expanding the design scope from supporting adolescents’ individual management decisions toward arranging mHealth core features as enablers for empowering adolescents, parents, and GPs to shift their focus from individual management toward reducing tensions via negotiation and shared decision-making, by enhancing communication, facilitating transition, and building alliances in the collaborative space.

### Comparison With Previous Studies

Systematic reviews describe how including mHealth apps during the treatment of adolescents with self-management needs from chronic conditions was associated with a host of observable benefits, which included positive changes in patients’ disease understanding, self-management capabilities, treatment adherence, and health behavior [25,27,29,32,66]. However, reviews with a clinical focus emphasized how understanding the patient’s disease-specific challenges and mHealth’s position within the treatment ecology is crucial for realizing the technologies’ potential [27,29] and leveraging patient-centered care [23]. Feasibility studies have documented how the efficacy of mobile and web-based interventions with tailored information, patient education, and self-directed exercises was similar to that of face-to-face consultations when treating knee pain in youths and adults [67]. However, these studies provided little insight about how adolescents integrated the information into their everyday management practices and beliefs, which was highlighted as important by GPs and young adults.

Qualitative studies by Slater et al [35,68,69] and Stinson et al [34,70] corroborated several of our findings about how mHealth-delivered health interventions (reminders, quantified self-tracking, and data feedback) held the potential for supporting adolescents with chronic pain through awareness, acceptance, and health decision-making between consultations, which could alleviate communicative obstacles during consultations. These studies focused on using mHealth data for enhanced communication during consultations as a driver for behavior change but provided little insights into how core features should accommodate the nonlinear, context-sensitive nature of mHealth interventions [38] or how to include parents as informal carers between GP consultations [71]. Systematic reviews by Moon et al [72] and Slater et al [68] corroborated participants’ visions about how mHealth could improve communication between GP clinics and home environments to facilitate collaborative care. However, both reviews highlight how adjusting apps to be integrated into formal and informal tasks and workflows of GPs and patients is crucial to ensure meaningfulness and continual use of mHealth and other digital patient education concepts.

### Adolescents’ Self-management as a Leveled Activity

Our analysis identified how adolescents’ management of their knee pain was a leveled activity, as described by Modi et al [73], which manifested within the individual and social domains. The descriptions obtained from young adults and GPs indicated a recognition of the knee pain as something processual (eg, a negative spiral), with parents and GPs being tasked with empowering adolescents to transition from a negative to a positive spiral with increased adaption, self-reflection, management, and control. These findings are similar to the observations made by Lerch and Thrane [74] in adolescents with other chronic conditions. The young adults’ descriptions indicated how adolescents’ main challenge was to balance managing their pain in situ, while simultaneously managing the social impacts of the knee pain [75]. Simultaneously, taking *action* to balance their knee pain allowed adolescents to explore, expand, and progress their self-management as illustrated by Johansen et al [56] and Cartwright et al [76]. This insight aligned with observations from Kralik et al [77] and Price [78] about how re-establishing balance or order in the wake of emerging symptoms acted as a point of learning for patients with chronic illness. Thus, we believe that a future mHealth app for adolescents with knee pain should target behaviors that re-establish an equilibrium with the knee pain, to enhance adolescents’ ability to facilitate their inquiry into their knee pain, which will improve their self-management in time.

### Core Features for Supporting Management Decisions

#### Providing Actionable Advice

Qualitative studies outline how managing knee pain is complex and involves adolescents balancing several activities including understanding their pain, maintaining function in everyday situations, care seeking, self-reflecting, and adjusting to a life with pain [56,59]. Our analysis identified targets for designing mHealth apps for supporting adolescents’ efforts toward managing their knee pain between consultations. For increasing adolescents’ understanding of pain, participants suggested incorporating a *first aid kit* feature, with information about the etiology of knee pain and actionable advice for pain alleviation. This would provide adolescents with reassurance, while promoting reflections about pain triggers and pain relief strategies, similar to what Rathleff et al [12] documented in clinical trials with their activity management tool.

#### Promoting Adherence

Regarding participating in care activities, participants highlighted how remembering and sustaining correct performance with exercises was a barrier, as observed by Faber et al [79] in youths. Participants suggested that incorporating a video feature with patient information, exercise instructions, and prompts for reminders could reduce maladaptive beliefs and empower exercise adherence. This aligns with the findings of Selhorst et al [80] and Holt et al [81], who showed that video-based handouts with patient education and exercise support limit maladaptive beliefs and boost adherence in youths with musculoskeletal pain. In addition, Riel et al [82] documented how live feedback increased the compliance of adolescents with knee pain with digital exercises. Studies exploring the use of gamification elements (leader boards, goals, minigames, leveling, esthetics, feedback, and rewards) to sustain long-term adherence to health interventions have shown positive effects such as enhancing medical adherence, disease understanding, and physical activity in adults and older people with chronic conditions [83-85]. Additional user-centered studies are needed to ensure that gamification elements align with adolescents’ needs to exert control of their knee pain [86] and are experienced as useful, meaningful, and inspiring for self-management in everyday contexts [83].

#### Enhancing Articulation

Finally, recall and articulation of knee pain were highlighted as barriers when seeking support from GPs and parents. Participants suggested that goal setting, quantified self-tracking, and pain journals could help adolescents in overcoming this barrier, by registering pain triggers, identifying pain thresholds, and assessing the value of behavior change. This resonated with the findings by Slater et al [69] about how adolescents were capable of setting personal recovery goals and the descriptions by Moon et al [72] and Lalloo et al [87] about quantified self-tracking's potential for enhancing adolescent-GP communication during clinical encounters, by allowing the adolescents to capture, explore, and articulate connections among management experiences, reflections, and outcomes [88]. However, our findings expanded upon this by illuminating the gap between ensuring that the self-tracked data are actionable to the adolescents and integrate into the GP’s decision-making process to facilitate mutual articulation. In total, our insights extend the current knowledge base and inform which individual management challenges should be addressed by mHealth core features in future mHealth concepts.

### mHealth Apps as Tools for Collaboration

#### Individual and Shared Processes

The young adults highlighted how GP and parental support had helped them to accept their knee pain and take on the role as explorers [34] and how experiencing having their requests rejected—real or perceived—increased doubts, dissatisfaction, and stagnation and prompted withdrawal, as observed in adults and youths with chronic pain [89-91]. Our analysis revealed how entering into treatment led to the emergence of a complex triadic relationship, where patients, parents, and GPs took on different roles, tasks, and responsibilities, similar to what Brooker [58] and Hohmann [57] outlined in the collaborative care triangle and what Kanstrup et al [92] presented as a complex interplay.

Nonetheless, our findings expanded upon this by describing how all participants were engaged in individual reflective processes and navigated both proximal and distal goals, as illustrated by Ryan and Sawin [93] in their family self-management concept. Furthermore, the emerging triadic relationship shared several properties with the *communities of practice* by Lave and Wenger [94] in terms of how participants organized, collaborated, and shared knowledge and how successful participation solidified the collaborative relationship. Ensuring that adolescents entered into an upward spiral acted as a shared domain of interest [95], with our analysis indicating how this acted as a cornerstone for shared health decision-making, as described by Makoul and Clayman [96]. Still, this required that said goals were articulated to be effective.

In terms of legitimate peripheral participation, our analysis outlined how adolescents were engaged in a *situated learning process*, which was driven by their explorative approach to managing knee pain [95]. In contrast, parents and GPs took on the roles as masters by observing, encouraging, diagnosing (GP), informing, educating (GP), and setting boundaries to facilitate the adolescents’ transition to self-management, as observed by Cha et al [43]. However, the absence of a shared vocabulary and repertoire for addressing self-management challenges made it difficult for participants to establish a working collaborative relationship. A future mHealth app should focus on creating a shared language for addressing the processual, proximal, and distal goals of participants [93] to support shared learning and avoid early treatment withdrawal.

#### Negotiation and Shared Decision-making

A key insight was how participants navigated the collaborative space through 2 modes of management behaviors, echoing the descriptions of Brooker [58] about members alternating between professionalism and providing care. Although adolescents, parents, and GPs navigated individual management challenges via their management decision-making, our matrix illustrated how participants solicited the expertise of other actors to overcome individual or contextual management barriers—an act that was collaborative in nature and required renegotiation of goals, roles, and tasks to succeed. Literature highlights that negotiation is an essential component in shared health decision-making [60,96]. Our matrix analysis identified how adolescents’ articulation, memory for exercises, parent’s knowledge, the long trajectory of knee pain, and GP’s ability to engage with adolescents inhibited negotiation and shared learning and increased tensions within the collaborative space. On the basis of these insights, our conceptual model presented 3 principles for designing or consolidating mHealth core features to act as enablers for mediating between participants to support negotiation, reduce tension, and create a basis for shared decision-making across multiple contexts. The model identified targets for enhancing the interpretive flexibility of core features to accommodate multiple user needs simultaneously [97] to enhance patient-GP communication, support parents in facilitating the adolescents’ transition, and help parents and GPs in building mutual trust and enhancing patient alliances.

#### Relations to Existing mHealth Concepts

Our exploration of mHealth literature related to this study failed to uncover mHealth concepts that incorporated all 3 principles for supporting negotiation and shared decision-making simultaneously; 3 designs were identified, which included 1 or 2 of the previously mentioned principles. The PainApp described by Koumpouros [98] supports patient-clinician communication, by using quantified self-tracking and aggregated data to empower carers’ clinical reasoning when *negotiating* treatments with adults with musculoskeletal pain. Furthermore, the PainApp uses self-tracking notifications as an *actionable* component to encourage patients to track and reflect on treatment effects at home. The ICanCope concept by Stinson et al [70] used a hybrid design to support adolescents with musculoskeletal pain to transition to adult care. The ICanCope app uses theory-informed interventions (self-tracking, goal setting, coping skills training, and social support) that are *actionable* to enhance adolescents’ symptom exploration and management *decision-making* at home. The web component included education features with self-tracked data to support adolescents’ articulation and features (discussion boards, goal sharing, and self-advocacy skills) that we interpret could enhance adolescents’ safety during patient-clinician communication and when negotiating roles and space with parents. As no mHealth tools were identified with features that directly supported parent-adolescent collaboration, the *diabetes management plan tool* [99] was included to exemplify how mHealth core features may bridge adolescents’ needs for safety from sanctions and parents’ needs to frame and facilitate their child’s self-management when making shared management decisions. By allowing users to choose and share items representing adolescents and parents’ tasks and obligations, the tool inspires role negotiation and shared decisions by articulating the tasks and responsibilities of both parties.

### Clinical and Design Implications

Literature describes how mHealth apps could act as a silver bullet for introducing patient-centered treatment approaches [23]. Our findings confirmed mHealth’s potential for augmenting collaborative care but illustrated how several bullets are required to leverage an mHealth apps’ utility as a tool for supporting shared decision-making when treating adolescent knee pain in general practice settings.

Several studies outlined mHealth’s potential for improving patient-clinician communication during consultations [35-37,68,72], but little knowledge exists on how mHealth apps could be used to build patient-GP relationships across time—a core theme in collaborative care [49]. Our analysis outlined how GPs took on the role as *change agent* during consultations, which entailed alternating between the *expert* and *guide* role to guide adolescents toward independence in management. However, our findings indicated that this process was dual reciprocal and required efforts from GPs to manage adolescents and parents’ expectations and biopsychosocial understanding of knee pain to alleviate collaborative tensions. Our analysis confirmed the observations by Brown et al [41] and Sawyer et al [9] about how adolescents’ articulation, ability to recall their pain developments, and memory for GP instructions acted as barriers to diagnosis, monitoring, adjusting treatments, and educating adolescents in managing their knee pain. mHealth apps with quantified self-tracked data could empower GPs to adapt measurement-based care when evaluating and adjusting interventions [36,72]. Furthermore, the act of tracking pain, reviewing aggregated data, and making management decisions could help adolescents to construct and articulate *theories* about how the knee pain progressed in time [88], which could then be discussed and qualified by the GP [36] to inform negotiation and shared decision-making [60]. Our analysis identified parents’ potential for taking on the role as informal carers and supporting the integration of treatments and management advice [71]; however, gaining the insight needed to know when to step in, set boundaries, and mediate between the adolescent and GPs required time, trust, and acceptance of the division of labor within the collaborative space.

The conceptual model outlined how adolescents, parents, and GPs required different modalities of information to sustain their roles, management practices, and inform negotiation. This places substantial demands on ensuring the interpretive flexibility of core features to act as enablers for shared decision-making. We infer that a future mHealth app should include 3 data loops: 1 with *actionable* small data interventions to support adolescents in exploring and balancing their knee pain [56]; 1 with static information for parents about etiology, red flags, and assistance for engaging with GPs; and 1 with aggregated mHealth data that allow GPs to *step into* the expert role and be effective in delivering treatments, adjusting treatments, and taking the *coach role* to provide self-management education. Ensuring that the app and information integrate into the ecologies of workflows, systems, and demands of general practice was crucial for achieving this effectiveness and should be explored further in future studies.

### Strengths and Limitations

The workshops’ inclusion of generative methods for facilitating dialogue and coconstruction of knowledge enabled us to extract the tacit and latent knowledge of our participants, which may not have been accessible via qualitative or focus group interviews [100]. However, by drawing upon the lived experiences of participants, the workshops were made vulnerable to recall and saliency bias [101,102]. Furthermore, having people working in groups may have made the process more open to *say-do* problems, compared with single-person interviews [103]. This was addressed by incorporating plenary discussions after the workshop phases and creating a pleasant atmosphere during the workshops [104]. A key strength was how each workshop followed the same design and used similar tools (case vignettes and inspiration cards) to facilitate discussions, which created a foundation for data synthesis. To avoid thematic reproduction, the design card’s themes were kept open and participants were encouraged to expand upon them throughout the workshops [105]. Although the workshops’ production of novel insights indicated that our efforts were successful, the extracted themes and models should be viewed as symbolic, ideal representations of the participants’ shared experience and should only serve to inform scientific inquiry. Despite our efforts to balance our workshop populations, the participants were predominantly women, which may have resulted in male-specific perspectives being overlooked during workshop discussions. Furthermore, no in-depth data were collected about the participants’ socioeconomic status, making it uncertain whether all potential user demographics have been represented. Literature highlights that the ideal number of participants for 1 workshop is between 8 and 16 [61], which we were able to accommodate in workshops 1 and 3. Despite the alternating sampling sizes, all workshops produced rich and descriptive data sets, with novel insights that could inform future mHealth tools. Thus, we did not interpret the low number of participants in workshop 2 as a limitation.

### Conclusions

mHealth apps are often hailed as a silver-bullet solution for introducing patient-centered and collaborative care approaches in complex care settings. Although actors navigated the complexity of the collaborative space through 2 modalities of management, role negotiation acted as a linchpin for reducing collaborative tensions and expanding actors’ management practices via shared decision-making. An mHealth app for treating adolescents with knee pain should accommodate multiple users and enable them to shift between individual management; take charge; and engage in negotiation of goals, roles, and tasks to inform shared decision-making. Our conceptual model identified 3 silver-bullet principles for consolidating mHealth core features as enablers for negotiation of goals, tasks, and roles by supporting patient-GP collaboration, empowering parents to facilitate transition, and cultivating the parent-GP alliance.

## Appendix

Multimedia Appendix 1 CONSORT-eHEALTH checklist (V 1.6.1).

Multimedia Appendix 2 Case vignette.

Multimedia Appendix 3 Examples of inspiration cards.

Multimedia Appendix 4 Overview of inspiration card themes.

Multimedia Appendix 5 Overview of steps taken during the data analysis.

Multimedia Appendix 6 Themes obtained from the thematic analysis.

Multimedia Appendix 7 Tension map.

Multimedia Appendix 8 Themes identified from plenary discussions.

## Abbreviations

CONSORT-EHEALTH: Consolidated Standards of Reporting Trials of Electronic and Mobile Health Applications and Online Telehealth
GP: general practitioner
mHealth: mobile health
RTA: reflexive thematic analysis
