# Supplementary material for: Exploring User Visions for Modeling mHealth Apps Toward Supporting Patient-Parent-Clinician Collaboration and Shared Decision-making When Treating Adolescent Knee Pain in General Practice: Workshop Study
Source: JMIR Hum Factors. 2023 Apr 28;10:e44462. doi: 10.2196/44462 (PMC10182461; doi:10.2196/44462)
Supplement: Multimedia Appendix 1 [file humanfactors_v10i1e44462_app1.pdf]

CONSORT-EHEALTH (V 1.6.1) - Submission/Publication Form

The CONSORT-EHEALTH checklist is intended for authors of randomized trials evaluating web-based and Internet-based applications/interventions, including mobile interventions, electronic games (incl multiplayer games), social media, certain telehealth applications, and other interactive and/or networked electronic applications. Some of the items (e.g. all subitems under item 5 - description of the intervention) may also be applicable for other study designs.

The goal of the CONSORT EHEALTH checklist and guideline is to be
a) a guide for reporting for authors of RCTs,
b) to form a basis for appraisal of an ehealth trial (in terms of validity)

CONSORT-EHEALTH items/subitems are MANDATORY reporting items for studies published in the Journal of Medical Internet Research and other journals / scientific societies endorsing the checklist.

Items numbered 1., 2., 3., 4a., 4b etc are original CONSORT or CONSORT-NPT (non-pharmacologic treatment) items.
Items with Roman numerals (i., ii, iii, iv etc.) are CONSORT-EHEALTH extensions/clarifications.

As the CONSORT-EHEALTH checklist is still considered in a formative stage, we would ask that you also RATE ON A SCALE OF 1-5 how important/useful you feel each item is FOR THE PURPOSE OF THE CHECKLIST and reporting guideline (optional).

Mandatory reporting items are marked with a red *.
In the textboxes, either copy & paste the relevant sections from your manuscript into this form - please include any quotes from your manuscript in QUOTATION MARKS,
or answer directly by providing additional information not in the manuscript, or elaborating on why the item was not relevant for this study.

YOUR ANSWERS WILL BE PUBLISHED AS A SUPPLEMENTARY FILE TO YOUR PUBLICATION IN JMIR AND ARE CONSIDERED PART OF YOUR PUBLICATION (IF ACCEPTED).
Please fill in these questions diligently. Information will not be copyedited, so please use proper spelling and grammar, use correct capitalization, and avoid abbreviations.

DO NOT FORGET TO SAVE AS PDF _AND_ CLICK THE SUBMIT BUTTON SO YOUR ANSWERS ARE IN OUR DATABASE !!!

Citation Suggestion (if you append the pdf as Appendix we suggest to cite this paper in the caption):
Eysenbach G, CONSORT-EHEALTH Group
[illegible]

Dit svar fylder for meget. Prøv at forkorte nogle af dine svar.

[illegible]

URL: http://www.jmir.org/2011/4/e126/
doi: 10.2196/jmir.1923
PMID: 22209829

smn.k.jhnsn@gmail.com (deles ikke) Skift konto

Kladden blev gemt

*Skal udfyldes

Your name *

First Last

Simon Kristoffer Johansen

Primary Affiliation (short), City, Country *

University of Toronto, Toronto, Canada

Aalborg University, Aalborg, Denmark

Your e-mail address *

abc@gmail.com

Skjohansen@dcm.aau.dk

Title of your manuscript *

Provide the (draft) title of your manuscript.

Searching for the Silver Bullet(s) - Exploring User Visions for Modelling MHealth Apps Towards Supporting Patient-Parent-Clinician Collaboration and Shared Decision-Making when Treating Adolescent Knee Pain in General Practice: A Workshop Study.

Dit svar fylder for meget. Prøv at forkorte nogle af dine svar.

Name of your App/Software/Intervention *

If there is a short and a long/alternate name, write the short name first and add the long name in brackets.

No software intervention was used.

Evaluated Version (if any)

e.g. "V1", "Release 2017-03-01", "Version 2.0.27913"

Not applicable

Language(s) *

What language is the intervention/app in? If multiple languages are available, separate by comma (e.g. "English, French")

Not applicable

URL of your Intervention Website or App

e.g. a direct link to the mobile app on app in appstore (itunes, Google Play), or URL of the website. If the intervention is a DVD or hardware, you can also link to an Amazon page.

Dit svar

URL of an image/screenshot (optional)

Dit svar

Dit svar fylder for meget. Prøv at forkorte nogle af dine svar.

Accessibility *

Can an enduser access the intervention presently?

- ○ access is free and open
- ○ access only for special usergroups, not open
- ○ access is open to everyone, but requires payment/subscription/in-app purchases
- ○ app/intervention no longer accessible
- ◉ Andet: Not applicable

Primary Medical Indication/Disease/Condition *

e.g. "Stress", "Diabetes", or define the target group in brackets after the condition, e.g. "Autism (Parents of children with)", "Alzheimers (Informal Caregivers of)"

Unspecified knee pain in adolescents (age 10-

Primary Outcomes measured in trial *

comma-separated list of primary outcomes reported in the trial

Patient-parent-clinician collaboration and shar

Secondary/other outcomes

Are there any other outcomes the intervention is expected to affect?

Enhanced capacity for management decision-making

Dit svar fylder for meget. Prøv at forkorte nogle af dine svar.

Recommended "Dose" *

What do the instructions for users say on how often the app should be used?

- ○ Approximately Daily
- ○ Approximately Weekly
- ○ Approximately Monthly
- ○ Approximately Yearly
- ○ "as needed"
- ◉ Andet: Not applicable

Approx. Percentage of Users (starters) still using the app as recommended after 3 months *

- ○ unknown / not evaluated
- ○ 0-10%
- ○ 11-20%
- ○ 21-30%
- ○ 31-40%
- ○ 41-50%
- ○ 51-60%
- ○ 61-70%
- ○ 71%-80%
- ○ 81-90%
- ○ 91-100%
- ◉ Andet: Not applicable

Dit svar fylder for meget. Prøv at forkorte nogle af dine svar.

Overall, was the app/intervention effective? *

- ○ yes: all primary outcomes were significantly better in intervention group vs control
- ○ partly: SOME primary outcomes were significantly better in intervention group vs control
- ○ no statistically significant difference between control and intervention
- ○ potentially harmful: control was significantly better than intervention in one or more outcomes
- ○ inconclusive: more research is needed
- ◉ Andet: Not applicable

Article Preparation Status/Stage *

At which stage in your article preparation are you currently (at the time you fill in this form)

- ○ not submitted yet - in early draft status
- ○ not submitted yet - in late draft status, just before submission
- ○ submitted to a journal but not reviewed yet
- ◉ submitted to a journal and after receiving initial reviewer comments
- ○ submitted to a journal and accepted, but not published yet
- ○ published
- ○ Andet:

Dit svar fylder for meget. Prøv at forkorte nogle af dine svar.

Journal *

If you already know where you will submit this paper (or if it is already submitted), please provide the journal name (if it is not JMIR, provide the journal name under "other")

- ○ not submitted yet / unclear where I will submit this
- ○ Journal of Medical Internet Research (JMIR)
- ○ JMIR mHealth and UHealth
- ○ JMIR Serious Games
- ○ JMIR Mental Health
- ○ JMIR Public Health
- ○ JMIR Formative Research
- ◉ Other JMIR sister journal
- ○ Andet:

Is this a full powered effectiveness trial or a pilot/feasibility trial? *

- ◉ Pilot/feasibility
- ○ Fully powered

Manuscript tracking number *

If this is a JMIR submission, please provide the manuscript tracking number under "other" (The ms tracking number can be found in the submission acknowledgement email, or when you login as author in JMIR. If the paper is already published in JMIR, then the ms tracking number is the four-digit number at the end of the DOI, to be found at the bottom of each published article in JMIR)

- ○ no ms number (yet) / not (yet) submitted to / published in JMIR
- ◉ Andet: 44462

Dit svar fylder for meget. Prøv at forkorte nogle af dine svar.

TITLE AND ABSTRACT

1a) TITLE: Identification as a randomized trial in the title

1a) Does your paper address CONSORT item 1a? *

I.e does the title contain the phrase "Randomized Controlled Trial"? (if not, explain the reason under "other")

- ○ yes
- ◉ Andet: No. The title does not contain the phrase "Randomized Controlled Tria

1a-i) Identify the mode of delivery in the title

Identify the mode of delivery. Preferably use “web-based” and/or “mobile” and/or “electronic game” in the title. Avoid ambiguous terms like “online”, “virtual”, “interactive”. Use “Internet-based” only if Intervention includes non-web-based Internet components (e.g. email), use “computer-based” or “electronic” only if offline products are used. Use “virtual” only in the context of “virtual reality” (3-D worlds). Use “online” only in the context of “online support groups”. Complement or substitute product names with broader terms for the class of products (such as “mobile” or “smart phone” instead of “iphone”), especially if the application runs on different platforms.

subitem not at all important

- 1 ○
- 2 ○
- 3 ○
- 4 ◉
- 5 ○

essential

Ryd markering

Dit svar fylder for meget. Prøv at forkorte nogle af dine svar.

Does your paper address subitem 1a-i? *

Copy and paste relevant sections from manuscript title (include quotes in quotation marks "like this" to indicate direct quotes from your manuscript), or elaborate on this item by providing additional information not in the ms, or briefly explain why the item is not applicable/relevant for your study

Yes and no. Our title uses the term mHealth app which signifies the use of a 'mobile health application', but does not include any of the above mentioned terms. As our focus was to leverage user input to formulate a design concept, taking an ambiguous approach to what the technology could be was nessecary which sould be reflected in the title. Thus we deem this non applicable to our study, but will reconsider if the editor is of a different opinion.

1a-ii) Non-web-based components or important co-interventions in title

Mention non-web-based components or important co-interventions in title, if any (e.g., “with telephone support”).

subitem not at all important

1 (selected)

2

3

4

5

essential

Ryd markering

Dit svar fylder for meget. Prøv at forkorte nogle af dine svar.

Does your paper address subitem 1a-ii?

Copy and paste relevant sections from manuscript title (include quotes in quotation marks "like this" to indicate direct quotes from your manuscript), or elaborate on this item by providing additional information not in the ms, or briefly explain why the item is not applicable/relevant for your study

While we used design-cards and use-cases during our future workshops, these were only included as part of the future workshop activities/interventions to guide and ensure continuity thoughout the workshops three phases. Thus we deem that the artifacts were less important then the intervention, and that this point is non-applicable to our study.

1a-iii) Primary condition or target group in the title

Mention primary condition or target group in the title, if any (e.g., “for children with Type I Diabetes”) Example: A Web-based and Mobile Intervention with Telephone Support for Children with Type I Diabetes: Randomized Controlled Trial

subitem not at all important

1 ○

2 ○

3 ○

4 ○

5 ◉

essential

Ryd markering

Dit svar fylder for meget. Prøv at forkorte nogle af dine svar.

Does your paper address subitem 1a-iii? *

Copy and paste relevant sections from manuscript title (include quotes in quotation marks "like this" to indicate direct quotes from your manuscript), or elaborate on this item by providing additional information not in the ms, or briefly explain why the item is not applicable/relevant for your study

We used the term "when Treating Adolescent Knee Pain in General Practice" in the title. We used knee pain as an overaching term which may include Partellofemoral Pain, Osgood Schlatter and Sinding-Larsen-Johansson Syndrome. This was relevant as qualitative studies have shown that patients consult their GPs mulitple time before they recive a diagnosis, and we intended our design to support their self-management and shared decision-making from they enter into general practice. No revisions.

1b) ABSTRACT: Structured summary of trial design, methods, results, and conclusions

NPT extension: Description of experimental treatment, comparator, care providers, centers, and blinding status.

1b-i) Key features/functionalities/components of the intervention and comparator in the METHODS section of the ABSTRACT

Mention key features/functionalities/components of the intervention and comparator in the abstract. If possible, also mention theories and principles used for designing the site. Keep in mind the needs of systematic reviewers and indexers by including important synonyms. (Note: Only report in the abstract what the main paper is reporting. If this information is missing from the main body of text, consider adding it)

subitem not at all important

1 ○

2 ○

3 ○

4 ◉

5 ○

essential

Ryd markering

Dit svar fylder for meget. Prøv at forkorte nogle af dine svar.

Does your paper address subitem 1b-i? *

Copy and paste relevant sections from the manuscript abstract (include quotes in quotation marks "like this" to indicate direct quotes from your manuscript), or elaborate on this item by providing additional information not in the ms, or briefly explain why the item is not applicable/relevant for your study

While our study focussed on identifying core-features and functionalities which were robust yet flexibile enough to support shared decision-making, we added an addtional sentence "- co-construction of knowledge and" (page 2, line 13) to outline how co-construction was the governing theoretical principle behind our intervention.

1b-ii) Level of human involvement in the METHODS section of the ABSTRACT

Clarify the level of human involvement in the abstract, e.g., use phrases like “fully automated” vs. “therapist/nurse/care provider/physician-assisted” (mention number and expertise of providers involved, if any). (Note: Only report in the abstract what the main paper is reporting. If this information is missing from the main body of text, consider adding it)

subitem not at all important

1 ○

2 ○

3 ○

4 ○

5 ◉

essential

Ryd markering

Dit svar fylder for meget. Prøv at forkorte nogle af dine svar.

Does your paper address subitem 1b-ii?

Copy and paste relevant sections from the manuscript abstract (include quotes in quotation marks "like this" to indicate direct quotes from your manuscript), or elaborate on this item by providing additional information not in the ms, or briefly explain why the item is not applicable/relevant for your study

Yes. As we applied a user-centered/participatory appraoch outlining how participants took part in the study becomes essential. We desiribed the process of the future workshops in these terms: "Three future workshops were conducted with young adults with chronic knee pain since adolescence, parents, and general practitioners. Each workshop followed similar procedures, utilizing case vignettes and design-cards to stimulate discussions,shared construction of knowledge and elicit visions for mHealth designs"Futhermore participants engagement in the workshop was described like this: "Nine young adults with chronic knee pain since adolescence, six parents and nine general practitioners participated in the workshops."

1b-iii) Open vs. closed, web-based (self-assessment) vs. face-to-face assessments in the METHODS section of the ABSTRACT

Mention how participants were recruited (online vs. offline), e.g., from an open access website or from a clinic or a closed online user group (closed usergroup trial), and clarify if this was a purely web-based trial, or there were face-to-face components (as part of the intervention or for assessment). Clearly say if outcomes were self-assessed through questionnaires (as common in web-based trials). Note: In traditional offline trials, an open trial (open-label trial) is a type of clinical trial in which both the researchers and participants know which treatment is being administered. To avoid confusion, use “blinded” or “unblinded” to indicated the level of blinding instead of “open”, as “open” in web-based trials usually refers to “open access” (i.e. participants can self-enrol). (Note: Only report in the abstract what the main paper is reporting. If this information is missing from the main body of text, consider adding it)

subitem not at all important

- 1 ○
- 2 ● (selected)
- 3 ○
- 4 ○
- 5 ○

essential

Dit svar fylder for meget. Prøv at forkorte nogle af dine svar.

Does your paper address subitem 1b-iii?

Copy and paste relevant sections from the manuscript abstract (include quotes in quotation marks "like this" to indicate direct quotes from your manuscript), or elaborate on this item by providing additional information not in the ms, or briefly explain why the item is not applicable/relevant for your study

Partially applicable. While our study didnt use questionaires to measure an effect, we used social media posts to identify young adults with knee pain and parents for inclusion, and cold calling for GPs. We revised our abstract to describe this better. Page 2, line 15 "Young adults and parents were included via social media posts targeting individuals in Northern Jutland. General Practitioners were included via email and cold calling."

The question of blinding is non-applicable to our study.

1b-iv) RESULTS section in abstract must contain use data

Report number of participants enrolled/assessed in each group, the use/uptake of the intervention (e.g., attrition/adherence metrics, use over time, number of logins etc.), in addition to primary/secondary outcomes. (Note: Only report in the abstract what the main paper is reporting. If this information is missing from the main body of text, consider adding it)

subitem not at all important

- 1 ○
- 2 ◉
- 3 ○
- 4 ○
- 5 ○

essential

Ryd markering

Dit svar fylder for meget. Prøv at forkorte nogle af dine svar.

Does your paper address subitem 1b-iv?

Copy and paste relevant sections from the manuscript abstract (include quotes in quotation marks "like this" to indicate direct quotes from your manuscript), or elaborate on this item by providing additional information not in the ms, or briefly explain why the item is not applicable/relevant for your study

Somewhat applicable. We identified how many participants took part in our workshops in this sentance. "Nine young adults with chronic knee pain since adolescence, six parents and nine general practitioners participated in the workshops" (Page 2, l. 18)

1b-v) CONCLUSIONS/DISCUSSION in abstract for negative trials

Conclusions/Discussions in abstract for negative trials: Discuss the primary outcome - if the trial is negative (primary outcome not changed), and the intervention was not used, discuss whether negative results are attributable to lack of uptake and discuss reasons. (Note: Only report in the abstract what the main paper is reporting. If this information is missing from the main body of text, consider adding it)

subitem not at all important

- 1 ○
- 2 ○
- 3 ○
- 4 ○
- 5 ◉

essential

Ryd markering

Dit svar fylder for meget. Prøv at forkorte nogle af dine svar.

Does your paper address subitem 1b-v?

Copy and paste relevant sections from the manuscript abstract (include quotes in quotation marks "like this" to indicate direct quotes from your manuscript), or elaborate on this item by providing additional information not in the ms, or briefly explain why the item is not applicable/relevant for your study

Yes. We described how our study identified a conceptual model for informing future mHealth designs as part of the results section: "The synthesis informed a conceptual model, outlining three principles for consolidating mHealth core-features as enablers for supporting role negotiation, to limit collaborative tensions and facilitating shared decision-making." (page 2, line 24). We also described the conclusion of this study "An mHealth app for treating adolescents with knee pain should be designed to accommodate multiple users, enabling them to shift between individual management decision-making, taking charge and engage in role negotiation to inform shared decision-making. We identified three silver-bullet principles for consolidating mHealth core-features as enablers for negotiation by supporting patient-GP collaboration, supporting transitions, and cultivating the parent-GP alliance."

INTRODUCTION

2a) In INTRODUCTION: Scientific background and explanation of rationale

Dit svar fylder for meget. Prøv at forkorte nogle af dine svar.

2a-i) Problem and the type of system/solution

Describe the problem and the type of system/solution that is object of the study: intended as stand-alone intervention vs. incorporated in broader health care program? Intended for a particular patient population? Goals of the intervention, e.g., being more cost-effective to other interventions, replace or complement other solutions? (Note: Details about the intervention are provided in “Methods” under 5)

subitem not at all important

1 ○

2 ○

3 ○

4 ◉

5 ○

essential

Ryd markering

Dit svar fylder for meget. Prøv at forkorte nogle af dine svar.

Does your paper address subitem 2a-i? *

Copy and paste relevant sections from the manuscript (include quotes in quotation marks "like this" to indicate direct quotes from your manuscript), or elaborate on this item by providing additional information not in the ms, or briefly explain why the item is not applicable/relevant for your study

Yes. Still our study is different because it exists in the space between general practice and patients home environments.

Used in relation to treatments:
A key question raised was how litterature describes how mHealth can potentially support adolescents in developing personal management strategies, and engage in co-care, but how there is little knowledge on how apps should be designed to do this. "Qualitative studies support that mHealth can assist the development of personal management strategies and assist young patients in engaging with clinicians in co-care situations [19,34,35]. Still, very few provide guidance on how future mHealth apps should be modelled to integrate into complex treatment settings as tools for enhancing existing treatments and facilitating continual care [36-38]." (Page. 3)

Intended patient population:
We focussed on adolescents with self-management needs due to knee pain, seeking treatment in general practice: "Self-management is essential for archiving recovery with knee pain [39]. Clinician-delivered patient education have been hailed as effective for teaching patients to self-manage their knee pain [13,40], yet health literacy studies point to adolescents here-and-now perspective on injuries, desire for independence and capacity for understanding GP instructions as barriers when supporting young patients [41,42]"

Aim. Supporting adolescents,parents and GP collaboration and shared decision-making.
We higlight how involved parents are important to support adolescents adherence to interventions between consultations. "Involved parents can aid adolescents transition into self-management, though task assistance, coaching, guidance, rewards and help during management mistakes [43-46] but this required agreement on tasks and responsibilities to enable collaboration [43-47]" which indicates that the setting is also in the everyday lives of patients. (Page 3).

"Shared decision-making holds the power to engage multiple stakeholders in the planning and facilitation of care, by merging patient and care-giver preferences with evidence-based practices [48] and the concept is central to the collaborative care process [49].]".

Dit svar fylder for meget. Prøv at forkorte nogle af dine svar.

2a-ii) Scientific background, rationale: What is known about the (type of) system

Scientific background, rationale: What is known about the (type of) system that is the object of the study (be sure to discuss the use of similar systems for other conditions/diagnoses, if appropiate), motivation for the study, i.e. what are the reasons for and what is the context for this specific study, from which stakeholder viewpoint is the study performed, potential impact of findings [2]. Briefly justify the choice of the comparator.

subitem not at all important

1 ○

2 ○

3 ○

4 ◉

5 ○

essential

Ryd markering

Does your paper address subitem 2a-ii? *

Copy and paste relevant sections from the manuscript (include quotes in quotation marks "like this" to indicate direct quotes from your manuscript), or elaborate on this item by providing additional information not in the ms, or briefly explain why the item is not applicable/relevant for your study

Not applicable as we didnt use an mHealth app during our intervention, and how our selection of methods was based on a knowledge gap which is only sparsely addressed in mHealth litterature. "Qualitative studies support that mHealth can assist the development of personal management strategies and assist young patients in engaging with clinicians in co-care situations [19,34,35]. Still, very few provide guidance on how future mHealth apps should be modelled to integrate into complex treatment settings as tools for enhancing existing treatments and facilitating continual care [36-38]." (Page 3.)

2b) In INTRODUCTION: Specific objectives or hypotheses

Dit svar fylder for meget. Prøv at forkorte nogle af dine svar.

Does your paper address CONSORT subitem 2b? *

Copy and paste relevant sections from the manuscript (include quotes in quotation marks "like this" to indicate direct quotes from your manuscript), or elaborate on this item by providing additional information not in the ms, or briefly explain why the item is not applicable/relevant for your study

Yes. The objectives of the study were as following: "The aim of this study was to identify principles for designing mHealth core-features which are robust enough to support adolescents' everyday management of their knee pain, and flexible enough to act as enablers for supporting patient-parent-GP collaboration and shared health decision-making. (page 4, line 5-9)

METHODS

3a) Description of trial design (such as parallel, factorial) including allocation ratio

Does your paper address CONSORT subitem 3a? *

Copy and paste relevant sections from the manuscript (include quotes in quotation marks "like this" to indicate direct quotes from your manuscript), or elaborate on this item by providing additional information not in the ms, or briefly explain why the item is not applicable/relevant for your study

Yes. "Action Research was included as a methodological framework to guide our application of methods, analysis, and knowledge production [50,51]. The projects intervention component consisted of three future workshops [52,53] one with young adults with knee pain since adolescence, one parents of adolescents with knee pain and one general practice physicians. Participants' dialogues were captured via audio recorders and analyzed separately via reflective thematic text analysis [54] to map the general challenges and visions for an mHealth application of each participant group. The extracted insights and visions were synthesized in a matrix, to identify lanes of collaboration, tension sources and facilitate the crystallization of design principles [55]" (Page 4, line 10)

3b) Important changes to methods after trial commencement (such as eligibility criteria), with reasons

Dit svar fylder for meget. Prøv at forkorte nogle af dine svar.

Does your paper address CONSORT subitem 3b? *

Copy and paste relevant sections from the manuscript (include quotes in quotation marks "like this" to indicate direct quotes from your manuscript), or elaborate on this item by providing additional information not in the ms, or briefly explain why the item is not applicable/relevant for your study

Not applicable. Despire this being a qualitative study there were no changes to the methods made during this study.

3b-i) Bug fixes, Downtimes, Content Changes

Bug fixes, Downtimes, Content Changes: ehealth systems are often dynamic systems. A description of changes to methods therefore also includes important changes made on the intervention or comparator during the trial (e.g., major bug fixes or changes in the functionality or content) (5-iii) and other “unexpected events” that may have influenced study design such as staff changes, system failures/downtimes, etc. [2].

subitem not at all important

- 1 (selected)
- 2
- 3
- 4
- 5

essential

Ryd markering

Does your paper address subitem 3b-i?

Copy and paste relevant sections from the manuscript (include quotes in quotation marks "like this" to indicate direct quotes from your manuscript), or elaborate on this item by providing additional information not in the ms, or briefly explain why the item is not applicable/relevant for your study

Not applicable, as our studys intervention component didnt include the use of digital or webbased tools.

Dit svar fylder for meget. Prøv at forkorte nogle af dine svar.

4a) Eligibility criteria for participants

Does your paper address CONSORT subitem 4a? *

Copy and paste relevant sections from the manuscript (include quotes in quotation marks "like this" to indicate direct quotes from your manuscript), or elaborate on this item by providing additional information not in the ms, or briefly explain why the item is not applicable/relevant for your study

Yes. Population one eligability: "Young adults (age 18-25) with longstanding recurring knee pain during adolescence (emerging age 10-15, duration more than 6 months), were included into study population one" and exclusion criteria "Exclusion criteria were competing musculoskeletal- or pain conditions unrelated to knee pain, long-term illness lasting more than 3 months, psychological issues which required medicine and surgery to the knee. Parents of adolescents with knee pain (emerging age 10-15) were included into study population two"

Population two eligibility criteria "Parents of adolescents with knee pain (emerging age 10-15) were included into study population two. Exclusion criteria´s were competing musculoskeletal- or pain conditions, severe physical handicaps, psychological issues, and surgery to the knee."

Population three "Inclusion criteria´s were employment in general practice for at least a year, experience in treating adolescent knee pain and willingness to participate. Participants for study population 1-2 were recruited trough social media posts targeting individuals in Northern Jutland, containing link to a form with questions relating to the inclusion criteria, contact information and consent forms."

Dit svar fylder for meget. Prøv at forkorte nogle af dine svar.

4a-i) Computer / Internet literacy

Computer / Internet literacy is often an implicit “de facto” eligibility criterion - this should be explicitly clarified.

subitem not at all important

1 (selected)

2

3

4

5

essential

Ryd markering

Does your paper address subitem 4a-i?

Copy and paste relevant sections from the manuscript (include quotes in quotation marks "like this" to indicate direct quotes from your manuscript), or elaborate on this item by providing additional information not in the ms, or briefly explain why the item is not applicable/relevant for your study

Not applicable. As our study didnt include a digital intervention participants digital litteracy was not deemed a factor in wheter they could partake/engage in the construction of knowledge during the workshops and provide their input in terms of what they found challanging in living with, parenting or treating adolescent knee pain.

Dit svar fylder for meget. Prøv at forkorte nogle af dine svar.

4a-ii) Open vs. closed, web-based vs. face-to-face assessments:

Open vs. closed, web-based vs. face-to-face assessments: Mention how participants were recruited (online vs. offline), e.g., from an open access website or from a clinic, and clarify if this was a purely web-based trial, or there were face-to-face components (as part of the intervention or for assessment), i.e., to what degree got the study team to know the participant. In online-only trials, clarify if participants were quasi-anonymous and whether having multiple identities was possible or whether technical or logistical measures (e.g., cookies, email confirmation, phone calls) were used to detect/prevent these.

subitem not at all important

1 ○

2 ○

3 ○

4 ○

5 ◉

essential

Ryd markering

Dit svar fylder for meget. Prøv at forkorte nogle af dine svar.

Does your paper address subitem 4a-ii? *

Copy and paste relevant sections from the manuscript (include quotes in quotation marks "like this" to indicate direct quotes from your manuscript), or elaborate on this item by providing additional information not in the ms, or briefly explain why the item is not applicable/relevant for your study

Yes. We described the workshop procedure in full detail in the manuscript (page 6, l. 14). "Workshops 1 and 2 (young adults and parents) were held at a local community center, while workshop 3 was held in the locals of Center for General Practice. All workshops lasted approximately 3 hours, distributed across three 40-minute phases and brakes. Each workshop was conducted with a primary coordinator (SKJ), a workshop facilitator who introduced workshop activities and guiding participants though the three phases and two co-facilitators (AMK, MSR, JLT) who would aid the facilitator in guiding group discussions and otherwise observe the process from the background. Upon arrival participants were divided into 3-4 person workgroups. Each workshop was initiated with a short introduction by the facilitator, and a presentation by an invited specialist; physiotherapist, mHealth specialist, eHealth specialist. The facilitator would then introduce the case vignettes, the inspiration cards corresponding to the given phase (see table 1) and provide instructions on how to complete the exercises of each phase. This procedure was repeated prior to each of the three phases (critique, ideation, and vision-phase) of the workshops. Each phase was concluded with a plenary discussion, were groups presented their toughs and ideas, while the facilitator summarized key points on a flipboard and asked follow-up questions. Upon completion of the final phase, all groups presented their visions for an mHealth application for feedback from other participants and facilitators. The workshops concluded with a debriefing, where participants were informed of their rights and filled in consent forms and were given the chance to ask final questions."

Dit svar fylder for meget. Prøv at forkorte nogle af dine svar.

4a-iii) Information giving during recruitment

Information given during recruitment. Specify how participants were briefed for recruitment and in the informed consent procedures (e.g., publish the informed consent documentation as appendix, see also item X26), as this information may have an effect on user self-selection, user expectation and may also bias results.

subitem not at all important

1 ○

2 ○

3 ○

4 ○

5 ◉

essential

Ryd markering

Does your paper address subitem 4a-iii?

Copy and paste relevant sections from the manuscript (include quotes in quotation marks "like this" to indicate direct quotes from your manuscript), or elaborate on this item by providing additional information not in the ms, or briefly explain why the item is not applicable/relevant for your study

Yes. "Potential participants, who expressed interest in participating and consented to contact, were contacted via phone, screened, provided informed about the project, participants rights and data treatment procedures (oral and written), and included. Participants in study population 3 were identified within Center for General Practice and Nord-KAP's clinician networks contacted via email and phone, informed, screened, and included into the project."

4b) Settings and locations where the data were collected

Dit svar fylder for meget. Prøv at forkorte nogle af dine svar.

Does your paper address CONSORT subitem 4b? *

Copy and paste relevant sections from the manuscript (include quotes in quotation marks "like this" to indicate direct quotes from your manuscript), or elaborate on this item by providing additional information not in the ms, or briefly explain why the item is not applicable/relevant for your study

Yes. "Workshops 1 and 2 (young adults and parents) were held at a local community center, while workshop 3 was held in the locals of Center for General Practice."

4b-i) Report if outcomes were (self-)assessed through online questionnaires

Clearly report if outcomes were (self-)assessed through online questionnaires (as common in web-based trials) or otherwise.

subitem not at all important

- 1 (selected)
- 2
- 3
- 4
- 5

essential

Ryd markering

Does your paper address subitem 4b-i? *

Copy and paste relevant sections from the manuscript (include quotes in quotation marks "like this" to indicate direct quotes from your manuscript), or elaborate on this item by providing additional information not in the ms, or briefly explain why the item is not applicable/relevant for your study

Not applicable. No outcomes were collected via online questionaires, web-based trails or otherwise.

Dit svar fylder for meget. Prøv at forkorte nogle af dine svar.

4b-ii) Report how institutional affiliations are displayed

Report how institutional affiliations are displayed to potential participants [on ehealth media], as affiliations with prestigious hospitals or universities may affect volunteer rates, use, and reactions with regards to an intervention.(Not a required item – describe only if this may bias results)

subitem not at all important

- 1 (selected)
- 2
- 3
- 4
- 5

essential

Ryd markering

Does your paper address subitem 4b-ii?

Copy and paste relevant sections from the manuscript (include quotes in quotation marks "like this" to indicate direct quotes from your manuscript), or elaborate on this item by providing additional information not in the ms, or briefly explain why the item is not applicable/relevant for your study

Not applicable. We did not use mHealth or eHealth media during our intervention.

5) The interventions for each group with sufficient details to allow replication, including how and when they were actually administered

Dit svar fylder for meget. Prøv at forkorte nogle af dine svar.

5-i) Mention names, credential, affiliations of the developers, sponsors, and owners

Mention names, credential, affiliations of the developers, sponsors, and owners [6] (if authors/evaluators are owners or developer of the software, this needs to be declared in a "Conflict of interest" section or mentioned elsewhere in the manuscript).

subitem not at all important

1 (selected)

2

3

4

5

essential

Ryd markering

Does your paper address subitem 5-i?

Copy and paste relevant sections from the manuscript (include quotes in quotation marks "like this" to indicate direct quotes from your manuscript), or elaborate on this item by providing additional information not in the ms, or briefly explain why the item is not applicable/relevant for your study

Not applicable. We did not use mHealth or eHealth media during the intervention of this study, and there were no developer, sponsors or owners involved in the study which may result in conflics of interest.

Dit svar fylder for meget. Prøv at forkorte nogle af dine svar.

5-ii) Describe the history/development process

Describe the history/development process of the application and previous formative evaluations (e.g., focus groups, usability testing), as these will have an impact on adoption/use rates and help with interpreting results.

subitem not at all important

1 (selected)

2

3

4

5

essential

Ryd markering

Does your paper address subitem 5-ii?

Copy and paste relevant sections from the manuscript (include quotes in quotation marks "like this" to indicate direct quotes from your manuscript), or elaborate on this item by providing additional information not in the ms, or briefly explain why the item is not applicable/relevant for your study

Not applicable. As we did not use mHealth or eHealth media during the intervention, no development process was conducted prior to the study.

Dit svar fylder for meget. Prøv at forkorte nogle af dine svar.

5-iii) Revisions and updating

Revisions and updating. Clearly mention the date and/or version number of the application/intervention (and comparator, if applicable) evaluated, or describe whether the intervention underwent major changes during the evaluation process, or whether the development and/or content was “frozen” during the trial. Describe dynamic components such as news feeds or changing content which may have an impact on the replicability of the intervention (for unexpected events see item 3b).

subitem not at all important

- 1 (selected)
- 2
- 3
- 4
- 5

essential

Ryd markering

Does your paper address subitem 5-iii?

Copy and paste relevant sections from the manuscript (include quotes in quotation marks "like this" to indicate direct quotes from your manuscript), or elaborate on this item by providing additional information not in the ms, or briefly explain why the item is not applicable/relevant for your study

Also not applicable. See above 5-i & 5-ii

Dit svar fylder for meget. Prøv at forkorte nogle af dine svar.

5-iv) Quality assurance methods

Provide information on quality assurance methods to ensure accuracy and quality of information provided [1], if applicable.

subitem not at all important

1 ○

2 ○

3 ○

4 ○

5 ◉

essential

Ryd markering

Does your paper address subitem 5-iv?

Copy and paste relevant sections from the manuscript (include quotes in quotation marks "like this" to indicate direct quotes from your manuscript), or elaborate on this item by providing additional information not in the ms, or briefly explain why the item is not applicable/relevant for your study

Yes. During the interventions we used plenary discussions to allow participants to evaluate each others opinions and envisioned solutions, which were guided by a facilitator. "Each phase was concluded with a plenary discussion, were groups presented their toughs and ideas, while the facilitator summarized key points on a flipboard and asked follow-up questions. Upon completion of the final phase, all groups presented their visions for an mHealth application for feedback from other participants and facilitators."

During the analysis we maintained a coding list and used discussions and stakeholder checks to ensure coding integrity and reach consensus in case of diverging findings. "Emerging thematic overlaps, divergencies and relationships identified within the individual analysis were discussed between the lead researcher (SKJ) and student workers (KH, VHS) until a consensus was reached." and "To ensure coding integrity, stakeholder checks were conducted by the lead researcher (SKJ) and student workers (KH, VHS), while coding list entries were discussed as the analysis progressed. The lead researcher (SKJ) was responsible for the final abstraction and presentation of findings in the five storybook themes which outlined the narrative in the results section."

Dit svar fylder for meget. Prøv at forkorte nogle af dine svar.

5-v) Ensure replicability by publishing the source code, and/or providing screenshots/screen-capture video, and/or providing flowcharts of the algorithms used

Ensure replicability by publishing the source code, and/or providing screenshots/screen-capture video, and/or providing flowcharts of the algorithms used. Replicability (i.e., other researchers should in principle be able to replicate the study) is a hallmark of scientific reporting.

subitem not at all important

- 1 (selected)
- 2
- 3
- 4
- 5

essential

Ryd markering

Does your paper address subitem 5-v?

Copy and paste relevant sections from the manuscript (include quotes in quotation marks "like this" to indicate direct quotes from your manuscript), or elaborate on this item by providing additional information not in the ms, or briefly explain why the item is not applicable/relevant for your study

Also not applicable. See above 5-i & 5-ii

Dit svar fylder for meget. Prøv at forkorte nogle af dine svar.

5-vi) Digital preservation

Digital preservation: Provide the URL of the application, but as the intervention is likely to change or disappear over the course of the years; also make sure the intervention is archived (Internet Archive, webcitation.org, and/or publishing the source code or screenshots/videos alongside the article). As pages behind login screens cannot be archived, consider creating demo pages which are accessible without login.

subitem not at all important

- 1 (selected)
- 2
- 3
- 4
- 5

essential

Ryd markering

Does your paper address subitem 5-vi?

Copy and paste relevant sections from the manuscript (include quotes in quotation marks "like this" to indicate direct quotes from your manuscript), or elaborate on this item by providing additional information not in the ms, or briefly explain why the item is not applicable/relevant for your study

Also not applicable. See above 5-i & 5-ii.

Dit svar fylder for meget. Prøv at forkorte nogle af dine svar.

5-vii) Access

Access: Describe how participants accessed the application, in what setting/context, if they had to pay (or were paid) or not, whether they had to be a member of specific group. If known, describe how participants obtained “access to the platform and Internet” [1]. To ensure access for editors/reviewers/readers, consider to provide a “backdoor” login account or demo mode for reviewers/readers to explore the application (also important for archiving purposes, see vi).

subitem not at all important

- 1 (selected)
- 2
- 3
- 4
- 5

essential

Ryd markering

Does your paper address subitem 5-vii? *

Copy and paste relevant sections from the manuscript (include quotes in quotation marks "like this" to indicate direct quotes from your manuscript), or elaborate on this item by providing additional information not in the ms, or briefly explain why the item is not applicable/relevant for your study

Also not applicable. See above 5-i & 5-ii.

Dit svar fylder for meget. Prøv at forkorte nogle af dine svar.

5-viii) Mode of delivery, features/functionalities/components of the intervention and comparator, and the theoretical framework

Describe mode of delivery, features/functionalities/components of the intervention and comparator, and the theoretical framework [6] used to design them (instructional strategy [1], behaviour change techniques, persuasive features, etc., see e.g., [7, 8] for terminology). This includes an in-depth description of the content (including where it is coming from and who developed it) [1]," whether [and how] it is tailored to individual circumstances and allows users to track their progress and receive feedback" [6]. This also includes a description of communication delivery channels and – if computer-mediated communication is a component – whether communication was synchronous or asynchronous [6]. It also includes information on presentation strategies [1], including page design principles, average amount of text on pages, presence of hyperlinks to other resources, etc. [1].

subitem not at all important

- 1 (selected)
- 2
- 3
- 4
- 5

essential

Ryd markering

Does your paper address subitem 5-viii? *

Copy and paste relevant sections from the manuscript (include quotes in quotation marks "like this" to indicate direct quotes from your manuscript), or elaborate on this item by providing additional information not in the ms, or briefly explain why the item is not applicable/relevant for your study

Also not applicable. See above 5-i & 5-ii.

Dit svar fylder for meget. Prøv at forkorte nogle af dine svar.

5-ix) Describe use parameters

Describe use parameters (e.g., intended "doses" and optimal timing for use). Clarify what instructions or recommendations were given to the user, e.g., regarding timing, frequency, heaviness of use, if any, or was the intervention used ad libitum.

subitem not at all important

- 1 (selected)
- 2
- 3
- 4
- 5

essential

Ryd markering

Does your paper address subitem 5-ix?

Copy and paste relevant sections from the manuscript (include quotes in quotation marks "like this" to indicate direct quotes from your manuscript), or elaborate on this item by providing additional information not in the ms, or briefly explain why the item is not applicable/relevant for your study

Also not applicable. See above 5-i & 5-ii.

Dit svar fylder for meget. Prøv at forkorte nogle af dine svar.

5-x) Clarify the level of human involvement

Clarify the level of human involvement (care providers or health professionals, also technical assistance) in the e-intervention or as co-intervention (detail number and expertise of professionals involved, if any, as well as “type of assistance offered, the timing and frequency of the support, how it is initiated, and the medium by which the assistance is delivered”. It may be necessary to distinguish between the level of human involvement required for the trial, and the level of human involvement required for a routine application outside of a RCT setting (discuss under item 21 – generalizability).

subitem not at all important

1 ○

2 ○

3 ○

4 ○

5 ◉

essential

Ryd markering

Dit svar fylder for meget. Prøv at forkorte nogle af dine svar.

Does your paper address subitem 5-x?

Copy and paste relevant sections from the manuscript (include quotes in quotation marks "like this" to indicate direct quotes from your manuscript), or elaborate on this item by providing additional information not in the ms, or briefly explain why the item is not applicable/relevant for your study

Yes. We described the procedure of the workshops in detail in the 'Settings and Procedure' section of the study.

"Each workshop was conducted with a primary coordinator (SKJ), a workshop facilitator who introduced workshop activities and guiding participants though the three phases and two co-facilitators (AMK, MSR, JLT) who would aid the facilitator in guiding group discussions and otherwise observe the process from the background. Upon arrival participants were divided into 3-4 person workgroups. Each workshop was initiated with a short introduction by the facilitator, and a presentation by an invited specialist; physiotherapist, mHealth specialist, eHealth specialist. The facilitator would then introduce the case vignettes, the inspiration cards corresponding to the given phase (see table 1) and provide instructions on how to complete the exercises of each phase. This procedure was repeated prior to each of the three phases (critique, ideation, and vision-phase) of the workshops. Each phase was concluded with a plenary discussion, were groups presented their toughs and ideas, while the facilitator summarized key points on a flipboard and asked follow-up questions. Upon completion of the final phase, all groups presented their visions for an mHealth application for feedback from other participants and facilitators. The workshops concluded with a debriefing, where participants were informed of their rights and filled in consent forms and were given the chance to ask final questions."

Dit svar fylder for meget. Prøv at forkorte nogle af dine svar.

5-xi) Report any prompts/reminders used

Report any prompts/reminders used: Clarify if there were prompts (letters, emails, phone calls, SMS) to use the application, what triggered them, frequency etc. It may be necessary to distinguish between the level of prompts/reminders required for the trial, and the level of prompts/reminders for a routine application outside of a RCT setting (discuss under item 21 – generalizability).

subitem not at all important

- 1 (selected)
- 2
- 3
- 4
- 5

essential

Ryd markering

Does your paper address subitem 5-xi? *

Copy and paste relevant sections from the manuscript (include quotes in quotation marks "like this" to indicate direct quotes from your manuscript), or elaborate on this item by providing additional information not in the ms, or briefly explain why the item is not applicable/relevant for your study

Not applicable as your study didnt involve an intervention using mobile or webbased solutions.

Dit svar fylder for meget. Prøv at forkorte nogle af dine svar.

5-xii) Describe any co-interventions (incl. training/support)

Describe any co-interventions (incl. training/support): Clearly state any interventions that are provided in addition to the targeted eHealth intervention, as ehealth intervention may not be designed as stand-alone intervention. This includes training sessions and support [1]. It may be necessary to distinguish between the level of training required for the trial, and the level of training for a routine application outside of a RCT setting (discuss under item 21 – generalizability.

subitem not at all important

1 (selected)

2

3

4

5

essential

Ryd markering

Does your paper address subitem 5-xii? *

Copy and paste relevant sections from the manuscript (include quotes in quotation marks "like this" to indicate direct quotes from your manuscript), or elaborate on this item by providing additional information not in the ms, or briefly explain why the item is not applicable/relevant for your study

Not applicable. As our study was a user-centered/design based study with future workshops, we didnt have a need for co-interventions or additional support.

6a) Completely defined pre-specified primary and secondary outcome measures, including how and when they were assessed

Dit svar fylder for meget. Prøv at forkorte nogle af dine svar.

Does your paper address CONSORT subitem 6a? *

Copy and paste relevant sections from the manuscript (include quotes in quotation marks "like this" to indicate direct quotes from your manuscript), or elaborate on this item by providing additional information not in the ms, or briefly explain why the item is not applicable/relevant for your study

No applicable. As our study was a user-centered/design based study with future workshops as its interventions we didnt define any primary or secondary outcome measures.

6a-i) Online questionnaires: describe if they were validated for online use and apply CHERRIES items to describe how the questionnaires were designed/deployed

If outcomes were obtained through online questionnaires, describe if they were validated for online use and apply CHERRIES items to describe how the questionnaires were designed/deployed [9].

subitem not at all important

1 (selected)

2

3

4

5

essential

Ryd markering

Does your paper address subitem 6a-i?

Copy and paste relevant sections from manuscript text

Not applicable. Our study didnt use any online questionaires.

Dit svar fylder for meget. Prøv at forkorte nogle af dine svar.

6a-ii) Describe whether and how “use” (including intensity of use/dosage) was defined/measured/monitored

Describe whether and how “use” (including intensity of use/dosage) was defined/measured/monitored (logins, logfile analysis, etc.). Use/adoption metrics are important process outcomes that should be reported in any ehealth trial.

subitem not at all important

1 (selected)

2

3

4

5

essential

Ryd markering

Does your paper address subitem 6a-ii?

Copy and paste relevant sections from manuscript text

Not applicable. The question of dosage was not compatable with future workshops.

Dit svar fylder for meget. Prøv at forkorte nogle af dine svar.

6a-iii) Describe whether, how, and when qualitative feedback from participants was obtained

Describe whether, how, and when qualitative feedback from participants was obtained (e.g., through emails, feedback forms, interviews, focus groups).

subitem not at all important

1 ○

2 ○

3 ○

4 ○

5 ◉

essential

Ryd markering

Does your paper address subitem 6a-iii?

Copy and paste relevant sections from manuscript text

Yes. Qualitative feedback on the intervention was obtained directly thoughout the workshop interventions, plenary discussions and during the debreifings after the workshops.

"The facilitator would then introduce the case vignettes, the inspiration cards corresponding to the given phase (see table 1) and provide instructions on how to complete the exercises of each phase. This procedure was repeated prior to each of the three phases (critique, ideation, and vision-phase) of the workshops. Each phase was concluded with a plenary discussion, were groups presented their toughs and ideas, while the facilitator summarized key points on a flipboard and asked follow-up questions. Upon completion of the final phase, all groups presented their visions for an mHealth application for feedback from other participants and facilitators" (page 6)

6b) Any changes to trial outcomes after the trial commenced, with reasons

Dit svar fylder for meget. Prøv at forkorte nogle af dine svar.

Does your paper address CONSORT subitem 6b? *

Copy and paste relevant sections from the manuscript (include quotes in quotation marks "like this" to indicate direct quotes from your manuscript), or elaborate on this item by providing additional information not in the ms, or briefly explain why the item is not applicable/relevant for your study

Not applicable. There was no changes to the trail outcomes after the study was commenced.

7a) How sample size was determined

NPT: When applicable, details of whether and how the clustering by care provides or centers was addressed

7a-i) Describe whether and how expected attrition was taken into account when calculating the sample size

Describe whether and how expected attrition was taken into account when calculating the sample size.

subitem not at all important

1 ○

2 ○

3 ○

4 ◉

5 ○

essential

Ryd markering

Dit svar fylder for meget. Prøv at forkorte nogle af dine svar.

Does your paper address subitem 7a-i?

Copy and paste relevant sections from manuscript title (include quotes in quotation marks "like this" to indicate direct quotes from your manuscript), or elaborate on this item by providing additional information not in the ms, or briefly explain why the item is not applicable/relevant for your study

Yes. "Furthermore, literature highlights between 8-16 as the ideal number of participants for one workshop [105] which we were able to accommodate in workshops one and three. Despite the alternating sampling sizes all workshops produced rich and descriptive datasets, with novel insights with could inform future mHealth tools. Thus, we did not interpret the low number of participants in workshop two as a limitation." (Page 23)

7b) When applicable, explanation of any interim analyses and stopping guidelines

Does your paper address CONSORT subitem 7b? *

Copy and paste relevant sections from the manuscript (include quotes in quotation marks "like this" to indicate direct quotes from your manuscript), or elaborate on this item by providing additional information not in the ms, or briefly explain why the item is not applicable/relevant for your study

Not applicable. As our intervention was not an ongoing process with progressive inclusion, we didnt conduct any interim analysis and didnt have any stopping guidelines.

8a) Method used to generate the random allocation sequence

NPT: When applicable, how care providers were allocated to each trial group

Does your paper address CONSORT subitem 8a? *

Copy and paste relevant sections from the manuscript (include quotes in quotation marks "like this" to indicate direct quotes from your manuscript), or elaborate on this item by providing additional information not in the ms, or briefly explain why the item is not applicable/relevant for your study

Not applicable. As our study did not use randomization, no random allocation sequence had to be generated.

Dit svar fylder for meget. Prøv at forkorte nogle af dine svar.

8b) Type of randomisation; details of any restriction (such as blocking and block size)

Does your paper address CONSORT subitem 8b? *

Copy and paste relevant sections from the manuscript (include quotes in quotation marks "like this" to indicate direct quotes from your manuscript), or elaborate on this item by providing additional information not in the ms, or briefly explain why the item is not applicable/relevant for your study

Not applicatble. As our study did not use randomization, no rarandomization strategy or blocking was used.

9) Mechanism used to implement the random allocation sequence (such as sequentially numbered containers), describing any steps taken to conceal the sequence until interventions were assigned

Does your paper address CONSORT subitem 9? *

Copy and paste relevant sections from the manuscript (include quotes in quotation marks "like this" to indicate direct quotes from your manuscript), or elaborate on this item by providing additional information not in the ms, or briefly explain why the item is not applicable/relevant for your study

Not applicable. As our study didnt use any form of randomization, no efforts had to be undertaken to conceal the process.

10) Who generated the random allocation sequence, who enrolled participants, and who assigned participants to interventions

Dit svar fylder for meget. Prøv at forkorte nogle af dine svar.

Does your paper address CONSORT subitem 10? *

Copy and paste relevant sections from the manuscript (include quotes in quotation marks "like this" to indicate direct quotes from your manuscript), or elaborate on this item by providing additional information not in the ms, or briefly explain why the item is not applicable/relevant for your study

Not applicable. As our study didnt use any form of randomization. The lead reasearcher SKJ was responsible for enrolling participants and assign them to the different workshops.

"Potential participants, who expressed interest in participating and consented to contact, were contacted via phone by SKJ, screened, provided informed about the project, participants rights and data treatment procedures (oral and written), and included. Participants in study population 3 were identified within Center for General Practice and Nord-KAP's clinician networks contacted via email and phone, informed, screened, and included into the project."

11a) If done, who was blinded after assignment to interventions (for example, participants, care providers, those assessing outcomes) and how

NPT: Whether or not administering co-interventions were blinded to group assignment

11a-i) Specify who was blinded, and who wasn't

Specify who was blinded, and who wasn't. Usually, in web-based trials it is not possible to blind the participants [1, 3] (this should be clearly acknowledged), but it may be possible to blind outcome assessors, those doing data analysis or those administering co-interventions (if any).

subitem not at all important

- 1 (selected)
- 2
- 3
- 4
- 5

essential

Dit svar fylder for meget. Prøv at forkorte nogle af dine svar.

Does your paper address subitem 11a-i? *

Copy and paste relevant sections from the manuscript (include quotes in quotation marks "like this" to indicate direct quotes from your manuscript), or elaborate on this item by providing additional information not in the ms, or briefly explain why the item is not applicable/relevant for your study

Not applicable. No blinding was used during our study.

11a-ii) Discuss e.g., whether participants knew which intervention was the "intervention of interest" and which one was the "comparator"

Informed consent procedures (4a-ii) can create biases and certain expectations - discuss e.g., whether participants knew which intervention was the "intervention of interest" and which one was the "comparator".

subitem not at all important

- 1 (selected)
- 2
- 3
- 4
- 5

essential

Ryd markering

Does your paper address subitem 11a-ii?

Copy and paste relevant sections from the manuscript (include quotes in quotation marks "like this" to indicate direct quotes from your manuscript), or elaborate on this item by providing additional information not in the ms, or briefly explain why the item is not applicable/relevant for your study

Not applicable. All workshop interventions were interventions of interest and were given equal wiight during the initial analysis.

Dit svar fylder for meget. Prøv at forkorte nogle af dine svar.

11b) If relevant, description of the similarity of interventions

(this item is usually not relevant for ehealth trials as it refers to similarity of a placebo or sham intervention to a active medication/intervention)

Does your paper address CONSORT subitem 11b? *

Copy and paste relevant sections from the manuscript (include quotes in quotation marks "like this" to indicate direct quotes from your manuscript), or elaborate on this item by providing additional information not in the ms, or briefly explain why the item is not applicable/relevant for your study

Yes. We described how all three workshop interventions followed the same approach to support comparison and synthesis of the extracted data. "Special care was taken to ensure that all three workshops followed the same procedure to heighten the compatibility of the extracted insights and visions." (Page 6).

12a) Statistical methods used to compare groups for primary and secondary outcomes

NPT: When applicable, details of whether and how the clustering by care providers or centers was addressed

Does your paper address CONSORT subitem 12a? *

Copy and paste relevant sections from the manuscript (include quotes in quotation marks "like this" to indicate direct quotes from your manuscript), or elaborate on this item by providing additional information not in the ms, or briefly explain why the item is not applicable/relevant for your study

Not applicable. We used thematic text analysis to analyze the data collected. No quantitative data was collected and no statistical analysis was conducted in this study.

Dit svar fylder for meget. Prøv at forkorte nogle af dine svar.

12a-i) Imputation techniques to deal with attrition / missing values

Imputation techniques to deal with attrition / missing values: Not all participants will use the intervention/comparator as intended and attrition is typically high in ehealth trials. Specify how participants who did not use the application or dropped out from the trial were treated in the statistical analysis (a complete case analysis is strongly discouraged, and simple imputation techniques such as LOCF may also be problematic [4]).

subitem not at all important

- 1 (selected)
- 2
- 3
- 4
- 5

essential

Ryd markering

Does your paper address subitem 12a-i? *

Copy and paste relevant sections from the manuscript (include quotes in quotation marks "like this" to indicate direct quotes from your manuscript), or elaborate on this item by providing additional information not in the ms, or briefly explain why the item is not applicable/relevant for your study

Not applicable. As we did not conduct a statistical analysis, no attrition or missing values were identified.

12b) Methods for additional analyses, such as subgroup analyses and adjusted analyses

Dit svar fylder for meget. Prøv at forkorte nogle af dine svar.

Does your paper address CONSORT subitem 12b? *

Copy and paste relevant sections from the manuscript (include quotes in quotation marks "like this" to indicate direct quotes from your manuscript), or elaborate on this item by providing additional information not in the ms, or briefly explain why the item is not applicable/relevant for your study

Somewhat applicable. The paper described how we conducted a thematic analysis in three sprints and used a matrix analysis to synthezise the data. "The data gathered during the three future workshops was analyzed through Reflexive Thematic Analysis (RTA) by Braun and Clarke [54] by the lead researcher (SKJ) and two student workers (KH, VHS)."

X26) REB/IRB Approval and Ethical Considerations [recommended as subheading under "Methods"] (not a CONSORT item)

X26-i) Comment on ethics committee approval

subitem not at all important

1 ○

2 ○

3 ○

4 ○

5 ◉

essential

Ryd markering

Dit svar fylder for meget. Prøv at forkorte nogle af dine svar.

Does your paper address subitem X26-i?

Copy and paste relevant sections from the manuscript (include quotes in quotation marks "like this" to indicate direct quotes from your manuscript), or elaborate on this item by providing additional information not in the ms, or briefly explain why the item is not applicable/relevant for your study

A study protocol was submitted for revisions by the regional board of Research Ethics in Northern Jutland who ruled the project was permitted to continue without registration based on national guidelines.

x26-ii) Outline informed consent procedures

Outline informed consent procedures e.g., if consent was obtained offline or online (how? Checkbox, etc.?), and what information was provided (see 4a-ii). See [6] for some items to be included in informed consent documents.

subitem not at all important

- 1 ○
- 2 ○
- 3 ○
- 4 ◉
- 5 ○

essential

Ryd markering

Does your paper address subitem X26-ii?

Copy and paste relevant sections from the manuscript (include quotes in quotation marks "like this" to indicate direct quotes from your manuscript), or elaborate on this item by providing additional information not in the ms, or briefly explain why the item is not applicable/relevant for your study

Yes. Conscent for data analysis was obtained during the debriefing after the final phase of the future workshops. "The workshops concluded with a debriefing, where participants were informed of their rights and filled in consent forms and were given the chance to ask final [illegible]

Dit svar fylder for meget. Prøv at forkorte nogle af dine svar.

X26-iii) Safety and security procedures

Safety and security procedures, incl. privacy considerations, and any steps taken to reduce the likelihood or detection of harm (e.g., education and training, availability of a hotline)

subitem not at all important

1 (selected)

2

3

4

5

essential

Ryd markering

Does your paper address subitem X26-iii?

Copy and paste relevant sections from the manuscript (include quotes in quotation marks "like this" to indicate direct quotes from your manuscript), or elaborate on this item by providing additional information not in the ms, or briefly explain why the item is not applicable/relevant for your study

Not applicable. Litterature on workshops descrtibes this method as a non-intrusive way of collecting data due to its short duration, and its non reliance on exercises, measurements as interventions. Thus, no measures were taken to reduce harm related to the intervention.

RESULTS

13a) For each group, the numbers of participants who were randomly assigned, received intended treatment, and were analysed for the primary outcome

NPT: The number of care providers or centers performing the intervention in each group and the number of patients treated by each care provider in each center

Dit svar fylder for meget. Prøv at forkorte nogle af dine svar.

Does your paper address CONSORT subitem 13a? *

Copy and paste relevant sections from the manuscript (include quotes in quotation marks "like this" to indicate direct quotes from your manuscript), or elaborate on this item by providing additional information not in the ms, or briefly explain why the item is not applicable/relevant for your study

Not applicable. No treatment was administered as part of this study.

13b) For each group, losses and exclusions after randomisation, together with reasons

Does your paper address CONSORT subitem 13b? (NOTE: Preferably, this is shown in a CONSORT flow diagram) *

Copy and paste relevant sections from the manuscript (include quotes in quotation marks "like this" to indicate direct quotes from your manuscript), or elaborate on this item by providing additional information not in the ms, or briefly explain why the item is not applicable/relevant for your study

Somewhat applicable. No randomizations was conducted and no participants were excluded after randomization. Still some participants failed to show up to the workshops or decided to cancel at last minute. "From the 11 included young adults, 9 participated (8 female; mean age 20, range 18-23) while 2 withdrew their participation." and "From the 11 parents included, 6 participated (5 females, mean age 46 years, range 41-52 years)." and "From the 12 included GP's, 9 participated (4 female, age 42 years mean, 30-63 years range) while 3 cancelled in advance."

Dit svar fylder for meget. Prøv at forkorte nogle af dine svar.

13b-i) Attrition diagram

Strongly recommended: An attrition diagram (e.g., proportion of participants still logging in or using the intervention/comparator in each group plotted over time, similar to a survival curve) or other figures or tables demonstrating usage/dose/engagement.

subitem not at all important

1 ○

2 ○

3 ◉

4 ○

5 ○

essential

Ryd markering

Does your paper address subitem 13b-i?

Copy and paste relevant sections from the manuscript or cite the figure number if applicable (include quotes in quotation marks "like this" to indicate direct quotes from your manuscript), or elaborate on this item by providing additional information not in the ms, or briefly explain why the item is not applicable/relevant for your study

Not applicable. As the study didnt involve that patients had to use a webbased or mobile device over time, we deem that no attrition diagram should be included.

14a) Dates defining the periods of recruitment and follow-up

Dit svar fylder for meget. Prøv at forkorte nogle af dine svar.

Does your paper address CONSORT subitem 14a? *

Copy and paste relevant sections from the manuscript (include quotes in quotation marks "like this" to indicate direct quotes from your manuscript), or elaborate on this item by providing additional information not in the ms, or briefly explain why the item is not applicable/relevant for your study

Not applicable. As our study didnt involve any type of follow-up after the workshop intervention.

14a-i) Indicate if critical "secular events" fell into the study period

Indicate if critical "secular events" fell into the study period, e.g., significant changes in Internet resources available or "changes in computer hardware or Internet delivery resources"

subitem not at all important

- 1 (selected)
- 2
- 3
- 4
- 5

essential

Ryd markering

Does your paper address subitem 14a-i?

Copy and paste relevant sections from the manuscript (include quotes in quotation marks "like this" to indicate direct quotes from your manuscript), or elaborate on this item by providing additional information not in the ms, or briefly explain why the item is not applicable/relevant for your study

Not applicable. No secular events fell during our intervention period.

Dit svar fylder for meget. Prøv at forkorte nogle af dine svar.

Does your paper address CONSORT subitem 14b? *

Copy and paste relevant sections from the manuscript (include quotes in quotation marks "like this" to indicate direct quotes from your manuscript), or elaborate on this item by providing additional information not in the ms, or briefly explain why the item is not applicable/relevant for your study

Not applicable. We didnt conduct a trail with an ongoining inclusion of participants.

15) A table showing baseline demographic and clinical characteristics for each group

NPT: When applicable, a description of care providers (case volume, qualification, expertise, etc.) and centers (volume) in each group

Does your paper address CONSORT subitem 15? *

Copy and paste relevant sections from the manuscript (include quotes in quotation marks "like this" to indicate direct quotes from your manuscript), or elaborate on this item by providing additional information not in the ms, or briefly explain why the item is not applicable/relevant for your study

Somewhat applicable. Due to the small number of participants (9 young adults, 6 parents and 9 general practioners) we described the baseline characteristics in the text prior to outlining the results of the study.

"The social media posts and phone screening generated 36 potential participants for workshop 1 (young adults) and 19 potential participants for workshop 2 (parents). Our efforts to contact GPs in the Northern Jutland area via emails and cold calling, yielded 17 potential participants from the 21 who were initially contacted (see model 1). From the 11 included young adults, 9 participated (8 female; mean age 20, range 18-23) while 2 withdrew their participation. All 9 participants in workshop 1 had experienced long-standing knee pain, emerging between age 11-16 years (age 13 years mean), and lasting for an average of 5,8 years (range, 3-9 years). From the 11 parents included, 6 participated (5 females, mean age 46 years, range 41-52 years). Parents reported, how all their adolescents (age 11 mean, range 10 - 12) had experienced knee pain for an average of 2 years (range 1-6 years) and how 4 of 6 (66%) of participants had participants had consulted their GP (66%), 2 of 6 (33%) had consulted a physiotherapists (33%), while 2 of 6 parents (33%) (33%) had yet not sought treatment for their child's knee pain. From the 12 included GP's, 9 participated (4 female, age 42 years mean, 30-63 years range) while 3 cancelled in advance. The participants in workshop 3 had an average of 8,5 years of experience from General Practice (range 1,5-25 years) with 7 of the 12 GP's (58%) reporting having a special interest in musculoskeletal conditions."

Dit svar fylder for meget. Prøv at forkorte nogle af dine svar.

15-i) Report demographics associated with digital divide issues

In ehealth trials it is particularly important to report demographics associated with digital divide issues, such as age, education, gender, social-economic status, computer/Internet/ehealth literacy of the participants, if known.

subitem not at all important

1 (selected)

2

3

4

5

essential

Ryd markering

Does your paper address subitem 15-i? *

Copy and paste relevant sections from the manuscript (include quotes in quotation marks "like this" to indicate direct quotes from your manuscript), or elaborate on this item by providing additional information not in the ms, or briefly explain why the item is not applicable/relevant for your study

No. As we were not doing a clinical trail but rather a workshop on developing a digital tool for supporting patients we didnt collect deep data on describing digital devides in our participant group as we considered participants lead-users. We have noted this as a limitation within the limiations section of the study.

16) For each group, number of participants (denominator) included in each analysis and whether the analysis was by original assigned groups

Dit svar fylder for meget. Prøv at forkorte nogle af dine svar.

16-i) Report multiple “denominators” and provide definitions

Report multiple “denominators” and provide definitions: Report N’s (and effect sizes) “across a range of study participation [and use] thresholds” [1], e.g., N exposed, N consented, N used more than x times, N used more than y weeks, N participants “used” the intervention/comparator at specific pre-defined time points of interest (in absolute and relative numbers per group). Always clearly define “use” of the intervention.

subitem not at all important

- 1 (selected)
- 2
- 3
- 4
- 5

essential

Ryd markering

Does your paper address subitem 16-i? *

Copy and paste relevant sections from the manuscript (include quotes in quotation marks "like this" to indicate direct quotes from your manuscript), or elaborate on this item by providing additional information not in the ms, or briefly explain why the item is not applicable/relevant for your study

Not applicable. The voice recordings from the workshops were transcribed and subjected to thematic text analysis which does not include reporting effects sizes.

Dit svar fylder for meget. Prøv at forkorte nogle af dine svar.

16-ii) Primary analysis should be intent-to-treat

Primary analysis should be intent-to-treat, secondary analyses could include comparing only “users”, with the appropriate caveats that this is no longer a randomized sample (see 18-i).

subitem not at all important

1 (selected)

2

3

4

5

essential

Ryd markering

Does your paper address subitem 16-ii?

Copy and paste relevant sections from the manuscript (include quotes in quotation marks "like this" to indicate direct quotes from your manuscript), or elaborate on this item by providing additional information not in the ms, or briefly explain why the item is not applicable/relevant for your study

Not applicable. Our scope of the study was to extract user visions for an mHealth application for supporting patients, parents and GPs shared decision-making during treatments. Thus, none of our analysis (TTA & Matrix) was conducted with the intention to treat anyone.

17a) For each primary and secondary outcome, results for each group, and the estimated effect size and its precision (such as 95% confidence interval)

Dit svar fylder for meget. Prøv at forkorte nogle af dine svar.

Does your paper address CONSORT subitem 17a? *

Copy and paste relevant sections from the manuscript (include quotes in quotation marks "like this" to indicate direct quotes from your manuscript), or elaborate on this item by providing additional information not in the ms, or briefly explain why the item is not applicable/relevant for your study

Not applicable. All the data we collected and analyzed was qualitative.

17a-i) Presentation of process outcomes such as metrics of use and intensity of use

In addition to primary/secondary (clinical) outcomes, the presentation of process outcomes such as metrics of use and intensity of use (dose, exposure) and their operational definitions is critical. This does not only refer to metrics of attrition (13-b) (often a binary variable), but also to more continuous exposure metrics such as “average session length”. These must be accompanied by a technical description how a metric like a “session” is defined (e.g., timeout after idle time) [1] (report under item 6a).

subitem not at all important

- 1 (selected)
- 2
- 3
- 4
- 5

essential

Ryd markering

Does your paper address subitem 17a-i?

Copy and paste relevant sections from the manuscript (include quotes in quotation marks "like this" to indicate direct quotes from your manuscript), or elaborate on this item by providing additional information not in the ms, or briefly explain why the item is not applicable/relevant for your study

Not applicable. As our study was qualitative and explorative in nature our findings couldnt

Dit svar fylder for meget. Prøv at forkorte nogle af dine svar.

17b) For binary outcomes, presentation of both absolute and relative effect sizes is recommended

Does your paper address CONSORT subitem 17b? *

Copy and paste relevant sections from the manuscript (include quotes in quotation marks "like this" to indicate direct quotes from your manuscript), or elaborate on this item by providing additional information not in the ms, or briefly explain why the item is not applicable/relevant for your study

Not applicable. Our study didnt present any abosulte or relative effectsizes

18) Results of any other analyses performed, including subgroup analyses and adjusted analyses, distinguishing pre-specified from exploratory

Does your paper address CONSORT subitem 18? *

Copy and paste relevant sections from the manuscript (include quotes in quotation marks "like this" to indicate direct quotes from your manuscript), or elaborate on this item by providing additional information not in the ms, or briefly explain why the item is not applicable/relevant for your study

Not applicable. Apart of the Thematic Text Analysis and Matrix analysis, the study didnt present any secondary or third analysis.

Dit svar fylder for meget. Prøv at forkorte nogle af dine svar.

18-i) Subgroup analysis of comparing only users

A subgroup analysis of comparing only users is not uncommon in ehealth trials, but if done, it must be stressed that this is a self-selected sample and no longer an unbiased sample from a randomized trial (see 16-iii).

subitem not at all important

- 1 (selected)
- 2
- 3
- 4
- 5

essential

Ryd markering

Does your paper address subitem 18-i?

Copy and paste relevant sections from the manuscript (include quotes in quotation marks "like this" to indicate direct quotes from your manuscript), or elaborate on this item by providing additional information not in the ms, or briefly explain why the item is not applicable/relevant for your study

Not applicable. As we were not conducting a trail there was no comparisons to other users or usual-care groups.

19) All important harms or unintended effects in each group

(for specific guidance see CONSORT for harms)

Dit svar fylder for meget. Prøv at forkorte nogle af dine svar.

Does your paper address CONSORT subitem 19? *

Copy and paste relevant sections from the manuscript (include quotes in quotation marks "like this" to indicate direct quotes from your manuscript), or elaborate on this item by providing additional information not in the ms, or briefly explain why the item is not applicable/relevant for your study

Not applicable. Litterature on workshops descrtibes this method as a non-intrusive way of collecting qualitative data due to its short duration and its non reliance on exercises, the method does not require us to screen participants for harms and uninteded effects from participation.

19-i) Include privacy breaches, technical problems

Include privacy breaches, technical problems. This does not only include physical "harm" to participants, but also incidents such as perceived or real privacy breaches [1], technical problems, and other unexpected/unintended incidents. "Unintended effects" also includes unintended positive effects [2].

subitem not at all important

1 (selected)

2

3

4

5

essential

Ryd markering

Does your paper address subitem 19-i?

Copy and paste relevant sections from the manuscript (include quotes in quotation marks "like this" to indicate direct quotes from your manuscript), or elaborate on this item by providing additional information not in the ms, or briefly explain why the item is not applicable/relevant for your study

Not applicable. There were no privacy breaches or technical problems identified during the [illegible]

Dit svar fylder for meget. Prøv at forkorte nogle af dine svar.

19-ii) Include qualitative feedback from participants or observations from staff/researchers

Include qualitative feedback from participants or observations from staff/researchers, if available, on strengths and shortcomings of the application, especially if they point to unintended/unexpected effects or uses. This includes (if available) reasons for why people did or did not use the application as intended by the developers.

subitem not at all important

1 ○

2 ○

3 ○

4 ○

5 ◉

essential

Ryd markering

Does your paper address subitem 19-ii?

Copy and paste relevant sections from the manuscript (include quotes in quotation marks "like this" to indicate direct quotes from your manuscript), or elaborate on this item by providing additional information not in the ms, or briefly explain why the item is not applicable/relevant for your study

Yes. We have included our qualitative feedback in the results section of the study. We will not post it here as it is too long.

DISCUSSION

22) Interpretation consistent with results, balancing benefits and harms, and considering other relevant evidence

NPT: In addition, take into account the choice of the comparator, lack of or partial blinding, and unequal expertise of care providers or centers in each group

Dit svar fylder for meget. Prøv at forkorte nogle af dine svar.

22-i) Restate study questions and summarize the answers suggested by the data, starting with primary outcomes and process outcomes (use)

Restate study questions and summarize the answers suggested by the data, starting with primary outcomes and process outcomes (use).

subitem not at all important

1 ○

2 ○

3 ○

4 ○

5 ◉

essential

Ryd markering

Dit svar fylder for meget. Prøv at forkorte nogle af dine svar.

Does your paper address subitem 22-i? *

Copy and paste relevant sections from the manuscript (include quotes in quotation marks "like this" to indicate direct quotes from your manuscript), or elaborate on this item by providing additional information not in the ms, or briefly explain why the item is not applicable/relevant for your study

Somewhat applicable. We added a summary of the primary outcomes/principal findings made during our study.
"Our findings revealed several key insights that needs to be considered when designing mHealth applications as tools for facilitating patient-centred treatment of adolescents with knee pain in general practice. Our analysis indicated how adolescents, parents and GPs entered a triadic relationship with different goals, tasks and information needs similar to what Hohmann [61] and Brooker [62] observed in paediatric settings. While all participants worked towards one outcome; ensuring that the adolescent entered an upwards spiral with decreasing pain and increasing control, adolescents actively facilitated this transition on an individual level, through their exploration of their knee pain in the present [56,63]. In contrast, parents and GP's roles were peripheral and focussed on supporting adolescents in navigating future management obstacles via observation, encouragement, boundary setting, and provisioning of management advice and information. Our analysis showed how all actors alternated between two modes of management behaviours. This included making individual management decisions to overcome contextual management challenges and engaging with other actors to utilize their expertise (as adolescents, parents, and GP's) to adjust management practices or collaborations; an act which involved a re-negotiating of goals, tasks, and responsibilities to be successful. While negotiation acted as a linchpin for shared decision making [64] our analysis identified how articulation, lack of knowledge, unfulfilled expectations and non-reciprocity inhibited negotiation and increased tensions in the collaborative space. Participants envisioned how an mHealth application for adolescents with knee pain should focus on providing reassurance, pattern recognition, and facilitate two-way communication. Our conceptual model identified three principles for expanding the design scope from supporting adolescents' individual management decisions, towards arranging mHealth core-features as enablers for empowering adolescents, parents, and GPs to shift their focus from individual management towards reducing tensions via negotiation and shared decision-making, by enhancing communication, facilitating transition, and building alliances in the collaborative space."

Dit svar fylder for meget. Prøv at forkorte nogle af dine svar.

22-ii) Highlight unanswered new questions, suggest future research

Highlight unanswered new questions, suggest future research.

subitem not at all important

1 ○

2 ○

3 ○

4 ○

5 ◉

essential

Ryd markering

Does your paper address subitem 22-ii?

Copy and paste relevant sections from the manuscript (include quotes in quotation marks "like this" to indicate direct quotes from your manuscript), or elaborate on this item by providing additional information not in the ms, or briefly explain why the item is not applicable/relevant for your study

Yes. Our analysis uncovered several new questions related to the implementation of mHealth applications in general practice setttings, which could be explored in future studies. "Ensuring that the app and information integrates into the ecologies of workflows, systems and demands of general practice was crucial for archiving this effectiveness and should be explored further in future studies."

20) Trial limitations, addressing sources of potential bias, imprecision, and, if relevant, multiplicity of analyses

Dit svar fylder for meget. Prøv at forkorte nogle af dine svar.

20-i) Typical limitations in ehealth trials

Typical limitations in ehealth trials: Participants in ehealth trials are rarely blinded. Ehealth trials often look at a multiplicity of outcomes, increasing risk for a Type I error. Discuss biases due to non-use of the intervention/usability issues, biases through informed consent procedures, unexpected events.

subitem not at all important

1 (selected)

2

3

4

5

essential

Ryd markering

Does your paper address subitem 20-i? *

Copy and paste relevant sections from the manuscript (include quotes in quotation marks "like this" to indicate direct quotes from your manuscript), or elaborate on this item by providing additional information not in the ms, or briefly explain why the item is not applicable/relevant for your study

Not applicable. We didnt conduct an ehealth trail, hence the bias sources are not applicabe to our study. Still we sought to identify bias sources like tunneling, conceptual circulation/reporduction and confirnmation bias during the analysis of data. "The data gathered during the three future workshops was analyzed through Reflexive Thematic Analysis (RTA) by Braun and Clarke [54] by the lead researcher (SKJ) and two student workers (KH, VHS).", "Emerging thematic overlaps, divergencies and relationships identified within the individual analysis were discussed between the lead researcher (SKJ) and student workers (KH, VHS) until a consensus was reached." and "To ensure coding integrity, stakeholder checks were conducted by the lead researcher (SKJ) and student workers (KH, VHS), while coding list entries were discussed as the analysis progressed. The lead researcher (SKJ) was responsible for the final abstraction and presentation of findings in the five storybook themes which outlined the narrative in the results section. All involved parties (SKJ, KH, VHS) approved the storybook themes, narrative, matrix analysis and conceptual model before the analysis was concluded."

Dit svar fylder for meget. Prøv at forkorte nogle af dine svar.

21) Generalisability (external validity, applicability) of the trial findings

NPT: External validity of the trial findings according to the intervention, comparators, patients, and care providers or centers involved in the trial

21-i) Generalizability to other populations

Generalizability to other populations: In particular, discuss generalizability to a general Internet population, outside of a RCT setting, and general patient population, including applicability of the study results for other organizations

subitem not at all important

- 1 (selected)
- 2
- 3
- 4
- 5

essential

Ryd markering

Does your paper address subitem 21-i?

Copy and paste relevant sections from the manuscript (include quotes in quotation marks "like this" to indicate direct quotes from your manuscript), or elaborate on this item by providing additional information not in the ms, or briefly explain why the item is not applicable/relevant for your study

Not applicable. As we conducted a qualitative study we cannot say with any degree of certainty that any of our findings can be generalized to other patient groups. Hence generalizabillity was not a success criteria when conducting this study.

Dit svar fylder for meget. Prøv at forkorte nogle af dine svar.

21-ii) Discuss if there were elements in the RCT that would be different in a routine application setting

Discuss if there were elements in the RCT that would be different in a routine application setting (e.g., prompts/reminders, more human involvement, training sessions or other co-interventions) and what impact the omission of these elements could have on use, adoption, or outcomes if the intervention is applied outside of a RCT setting.

subitem not at all important

- 1 (selected)
- 2
- 3
- 4
- 5

essential

Ryd markering

Does your paper address subitem 21-ii?

Copy and paste relevant sections from the manuscript (include quotes in quotation marks "like this" to indicate direct quotes from your manuscript), or elaborate on this item by providing additional information not in the ms, or briefly explain why the item is not applicable/relevant for your study

Not applicable. We did not conduct a trail or an RCT.

OTHER INFORMATION

23) Registration number and name of trial registry

Dit svar fylder for meget. Prøv at forkorte nogle af dine svar.

Does your paper address CONSORT subitem 23? *

Copy and paste relevant sections from the manuscript (include quotes in quotation marks "like this" to indicate direct quotes from your manuscript), or elaborate on this item by providing additional information not in the ms, or briefly explain why the item is not applicable/relevant for your study

Not applicable. "A study protocol was submitted for revisions by the regional board of Research Ethics in Northern Jutland who ruled the project was permitted to continue without registration based on national guidelines." Hence no registration number/name of trail registry was provided.

24) Where the full trial protocol can be accessed, if available

Does your paper address CONSORT subitem 24? *

Cite a Multimedia Appendix, other reference, or copy and paste relevant sections from the manuscript (include quotes in quotation marks "like this" to indicate direct quotes from your manuscript), or elaborate on this item by providing additional information not in the ms, or briefly explain why the item is not applicable/relevant for your study

Not applicable. As this was a qualitative study, no full trail protocol was uploaded to any repository prior to undertaking the study.

25) Sources of funding and other support (such as supply of drugs), role of funders

Does your paper address CONSORT subitem 25? *

Copy and paste relevant sections from the manuscript (include quotes in quotation marks "like this" to indicate direct quotes from your manuscript), or elaborate on this item by providing additional information not in the ms, or briefly explain why the item is not applicable/relevant for your study

The research presented in this article is independent work, which is supported and financed by the Research Foundation for General Practice (A1819, Simon Kristoffer Johansen). The study was conducted at Center for General Practice (CAM-AAU) Department of Clinical Medicine at Aalborg University.

Dit svar fylder for meget. Prøv at forkorte nogle af dine svar.

X27) Conflicts of Interest (not a CONSORT item)

X27-i) State the relation of the study team towards the system being evaluated

In addition to the usual declaration of interests (financial or otherwise), also state the relation of the study team towards the system being evaluated, i.e., state if the authors/evaluators are distinct from or identical with the developers/sponsors of the intervention.

subitem not at all important

- 1 ○
- 2 ○
- 3 ○
- 4 ○
- 5 ◉

essential

Ryd markering

Does your paper address subitem X27-i?

Copy and paste relevant sections from the manuscript (include quotes in quotation marks "like this" to indicate direct quotes from your manuscript), or elaborate on this item by providing additional information not in the ms, or briefly explain why the item is not applicable/relevant for your study

Not applicable as we didnt use any application/systems within this study.

About the CONSORT EHEALTH checklist

Dit svar fylder for meget. Prøv at forkorte nogle af dine svar.

As a result of using this checklist, did you make changes in your manuscript? *

- ○ yes, major changes
- ◉ yes, minor changes
- ○ no

What were the most important changes you made as a result of using this checklist?

We identifed how the clinical characteristics of participants did not include descriptions of participants socio-economic backgrounds. This was noted as a limitation and led us to update the limitation section of our study.

How much time did you spend on going through the checklist INCLUDING making changes in your manuscript *

We used six hours in total to fill out this questionaire.

As a result of using this checklist, do you think your manuscript has improved? *

- ◉ yes
- ○ no
- ○ Andet:

Dit svar fylder for meget. Prøv at forkorte nogle af dine svar.

Would you like to become involved in the CONSORT EHEALTH group?

This would involve for example becoming involved in participating in a workshop and writing an "Explanation and Elaboration" document

- ○ yes
- ● no
- ○ Andet:

Ryd markering

Any other comments or questions on CONSORT EHEALTH

We have no comments at this given time.

STOP - Save this form as PDF before you click submit

To generate a record that you filled in this form, we recommend to generate a PDF of this page (on a Mac, simply select "print" and then select "print as PDF") before you submit it.

When you submit your (revised) paper to JMIR, please upload the PDF as supplementary file.

Don't worry if some text in the textboxes is cut off, as we still have the complete information in our database. Thank you!

Final step: Click submit !

Click submit so we have your answers in our database!

Send Ryd formular

Indsend aldrig adgangskoder via Google Analyse.

Dette indhold er hverken oprettet eller godkendt af Google. Rapportér misbrug - Servicevilkår - Privatlivspolitik

Google Analyse

Dit svar fylder for meget. Prøv at forkorte nogle af dine svar.

Dit svar fylder for meget. Prøv at forkorte nogle af dine svar.
