# Supplementary material for: Exploring User Visions for Modeling mHealth Apps Toward Supporting Patient-Parent-Clinician Collaboration and Shared Decision-making When Treating Adolescent Knee Pain in General Practice: Workshop Study
Source: JMIR Hum Factors. 2023 Apr 28;10:e44462. doi: 10.2196/44462 (PMC10182461; doi:10.2196/44462)
Supplement: Multimedia Appendix 2 [file humanfactors_v10i1e44462_app2.pdf]

Appendix 2 – Case vignette; Frederikke.

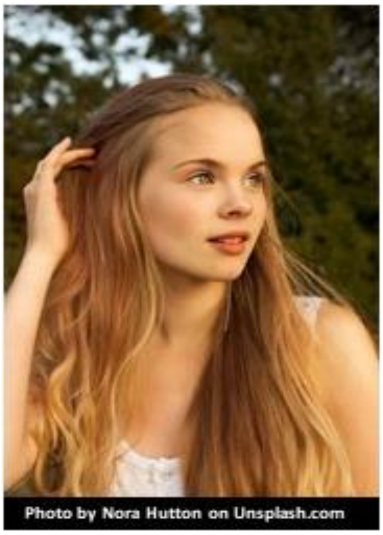

Case, Frederikke.

Frederikke just turned 15 years old. She was born in Aalborg but grew up in Noerresundby (a suburb) where she lives with her father, mother, and younger brother. She is currently in the 9th grade, where she is getting 'okay' grades, and even though she really dislikes the concept of homework. Frederikke has always been active, but during recent years, handball has started taking up more of her free time. This is especially true after she was moved to the A team. Several of her friends also play handball, and in between matches and training Frederikke would sometimes go to the handball club to hang out with her friends.

During the last 3-4 months Frederikke has started to notice something was off with her knees. It began as a strange sensation which emerged when climbing stairs, but quickly disappeared again. Later she started noticing a slight tenderness or stiffness in her knees during training – but this often subsided as she was warmed up. Frederikke has had small injuries before – sprained fingers and thigh contusions from impacts during matches – which she consulted her GP for. Yet this felt different. Still, as she was able to keep up with her teammates, Frederikke reckoned there was no cause for alarm, but she did mention it to her parents who told her it was likely to be growing pains.

During the last couple of months, Frederikke have been experiencing a buzzing or throbbing sensation after handball training, and sometimes she has had to go lie down on her bed to cope with it. Apart from this, Frederikke has experienced what she describes as her knees 'giving up' during handball training sessions. This is painful and Frederikke have had to lower her tempo to keep on playing. While this is frustrating to her, she hasn't told her coach or team mates yet, as she is nervous that they might think that she is being whiney, lazy, trying to get attention or they might take her off the team. She did ask her mother whether it was normal to have pain in her knees, and they have agreed to see her doctor about it.

Frederikke is looking forward to going to a sports boarding school in 10th grade, but she is worried if this is possible now her knees are acting up.

Appendix 1: The case vignette used to facilitate participants discussions during each workshop along translated to English. The case was developed in collaboration with General practitioners, physiotherapists, parents of adolescents with knee pain and young adults with knee pain emerging during adolescence.
