# Supplementary material for: Exploring User Visions for Modeling mHealth Apps Toward Supporting Patient-Parent-Clinician Collaboration and Shared Decision-making When Treating Adolescent Knee Pain in General Practice: Workshop Study
Source: JMIR Hum Factors. 2023 Apr 28;10:e44462. doi: 10.2196/44462 (PMC10182461; doi:10.2196/44462)
Supplement: Multimedia Appendix 3 [file humanfactors_v10i1e44462_app3.pdf]

Appendix 3 – Examples Inspiration cards.

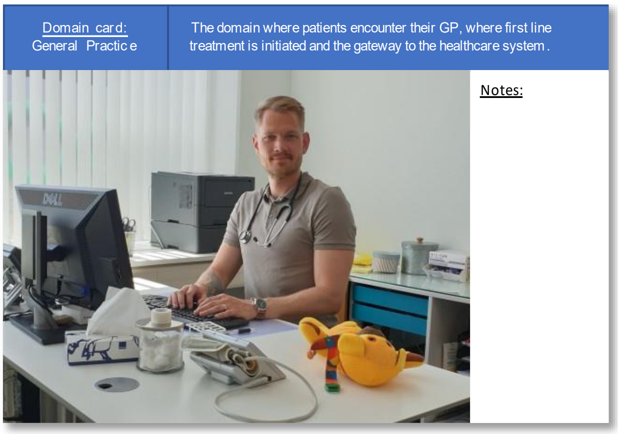

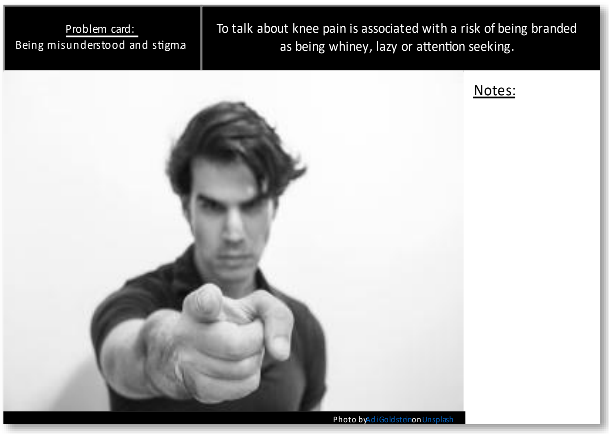

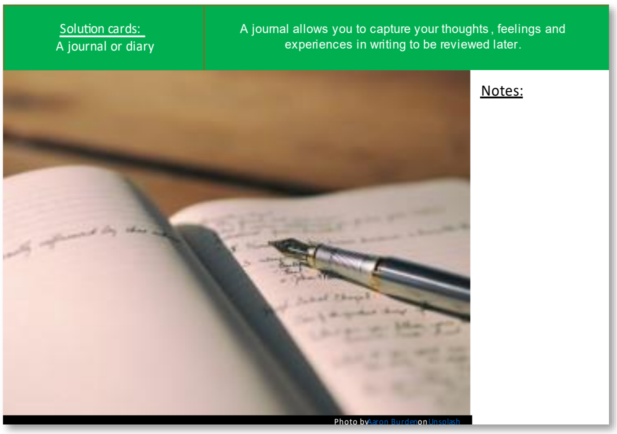

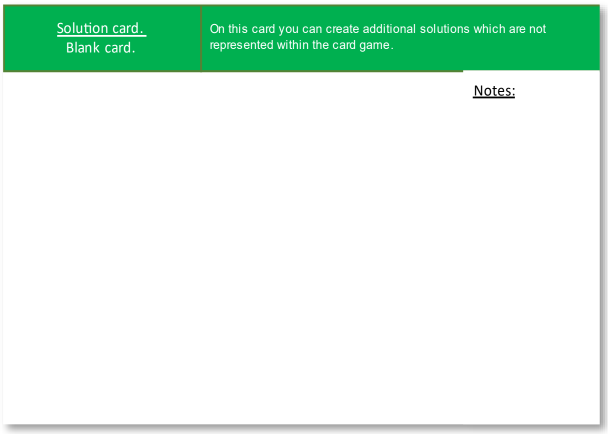

Appendix 2 (above): The workshops were conducted with three sets of inspiration cards which illustrated different domains where adolescents experienced knee pain (blue, domain cards), emerging problems or challenges (black, problem cards) and solutions (green, solution cards) with could help adolescents, parents, or GPs to resolve pain or pain related challenges. Furthermore, several additional blank cards were added to each set of card games to allow participants to add domains, themes or solutions which were not represented in the card game. The inspiration cards were designed and tested for comprehensibility and relevance with GP's, Physiotherapists, Parents of adolescents with knee pain and young adults with knee pain.

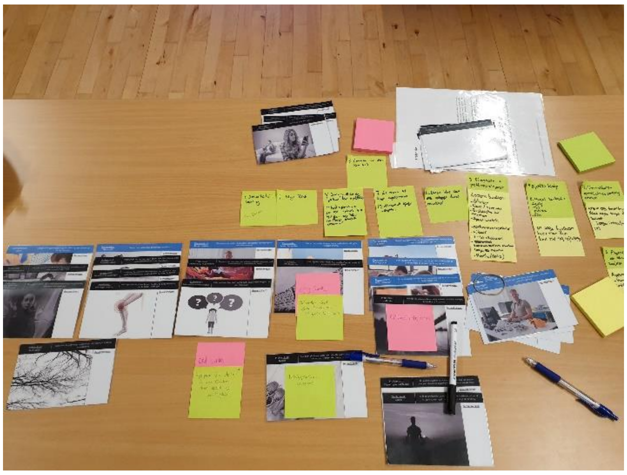

Appendix 2 (below): The pictures illustrated how participants used the different cards to explore connections between themes and used this as a facilitator for discussions. Apart from writing on the cards, participants were given post-its (red, problems; green, solutions; yellow, visions) where they could note and capture identified problems, ideas for solutions or visions for an mHealth app as they emerged during the discussions.
