# Supplementary material for: Exploring User Visions for Modeling mHealth Apps Toward Supporting Patient-Parent-Clinician Collaboration and Shared Decision-making When Treating Adolescent Knee Pain in General Practice: Workshop Study
Source: JMIR Hum Factors. 2023 Apr 28;10:e44462. doi: 10.2196/44462 (PMC10182461; doi:10.2196/44462)
Supplement: Multimedia Appendix 4 [file humanfactors_v10i1e44462_app4.pdf]

Appendix 4 – Overview of inspiration card themes.

Overview of inspiration card themes.	
**Domain cards.**	The GPs clinic.<br>The home.<br>The school.<br>During sports participation.<br>With friends.<br>Other interests.<br>Other types of treatment.<br>Blank cards.
**Problem cards.**	Patient-GP communication.<br>The adolescents' theories/beliefs about the knee pain.<br>Exercises.<br>Forgetting the pain.<br>You're not the boss of me!<br>The adolescents pain experience.<br>Motivation.<br>Finding the limit.<br>Information from external sources.<br>Parental involvement.<br>Acceptance.<br>Being misunderstood and stigma.<br>Insecurity<br>Parents expectations.<br>Explaining the knee pain.<br>Blank cards.
**Solution cards.**	A journal or diary.<br>A checklist.<br>A scale for measuring knee pain.<br>More time with a healthcare provider.<br>A book on knee pain.<br>A support group.<br>Patient stories.<br>A tool for visualizing the knee pain.<br>A first aid kit.<br>Exercise instructions.

Appendix 3: A overview of all themes represented within the inspiration card games organized in relation to whether themes represented domains, problems or challenges or possible solutions.
