# Supplementary material for: Exploring User Visions for Modeling mHealth Apps Toward Supporting Patient-Parent-Clinician Collaboration and Shared Decision-making When Treating Adolescent Knee Pain in General Practice: Workshop Study
Source: JMIR Hum Factors. 2023 Apr 28;10:e44462. doi: 10.2196/44462 (PMC10182461; doi:10.2196/44462)
Supplement: Multimedia Appendix 5 [file humanfactors_v10i1e44462_app5.pdf]

Appendix 5 – Overview of the steps within the reflexive thematic analysis of workshop data.

**Analytical steps.**	**Step 1: Familiarization.**	**Step 2: Coding.**	**Step 3: Condensation & identification of themes.**	**Step 4: Synthesis.**
**Analytical process:**	Research prepares the raw datasets for analysis, familiarizing themselves with the contents.	Identification of units of meaning through vertical readings of texts. Formation of themes, main- and subthemes.	Condensation of texts trough horizontal readings, renaming and refining of themes. Themes and subthemes were merged to eliminate overlaps and redundancies.	Merger of the condensed themes across all three analyses. Extracting a shared narrative and identifying design principles.
**Analysis performed by:**	Audio recordings were transcribed for meaning retention, by SKJ, VHS and KH. Researchers reviewed the themes identified during the workshops plenary discussions to identify points of interest within the text. In-depth readthroughs were conducted by all to gain familiarity with the texts.	Themes were identified, named, and labelled according to themes by SKJ, VHS and KH through several rounds of coding. Emerging codes were revised, merged, and expanded as new insights were extracted from the data.	Researchers (SKJ, VHS, KH) condensed the selected themes into summary form. Identification and elimination of thematic overlaps within each individual analysis. Themes were merged and renamed as the refinement process progressed.<br><br>The coding lists were updated and maintained as the process progressed.	Condensed texts from all three analysis were merged to identify 5 storybook themes which formed a combined narrative.<br><br>Simultaneously, the content of themes (step 3) was organized in a framework by SKJ, outlined challenges, collaborative pathways and identified three design principles for designing mHealth core-features as enablers for collaboration and shared decision-making.
**Analytical tools:**	Express scribe*.	NVivo**, mind maps and coding lists.	NVivo**, Word***, coding lists.	Word***
**Analytical object:**	None.	Workshop 1; 10 main themes, 34 themes and 3 subthemes.<br>Workshop 2; 4 main themes, 17 themes and 13 subthemes.<br>Work 3; 5 main themes, 21 themes and 8 subthemes.	Workshop 1: 8 themes.<br>Workshop 2: 9 themes.<br>Workshop 3: 7 themes.	A narrative of 5 storybook themes and a conceptual model of collaborative pathways.
**Evaluation:**	None.	Coded texts were switched between research (SKJ, KH, VHS) and checked to ensure the validity of the coding.	Extracted condensates were shared and discussed between researchers (SKJ, KH, VHS), thus ensuring all relevant topics were covered.	Evaluation and feedback from VHS and KH.

***Express Scribe v. 7.01 (NCH software, Canberra, Australia)**
****NVivo 11 v. 11.01.4.11 (QSR International Pty Ltd, Melbourne, Australia)**
*****Microsoft Word for Office 365 MSO (Microsoft office, Richmond, USA)**
