# Supplementary material for: Exploring User Visions for Modeling mHealth Apps Toward Supporting Patient-Parent-Clinician Collaboration and Shared Decision-making When Treating Adolescent Knee Pain in General Practice: Workshop Study
Source: JMIR Hum Factors. 2023 Apr 28;10:e44462. doi: 10.2196/44462 (PMC10182461; doi:10.2196/44462)
Supplement: Multimedia Appendix 6 [file humanfactors_v10i1e44462_app6.pdf]

# Appendix 6 – Themes identified during the reflexive thematic analysis.

| Workshop 1. Adolescents | Workshop 2. Parents | Workshop 3. GP's |
|---|---|---|
| **The everyday experience of knee pain (start).** | **The parents experience of the adolescents' knee pain. (start)** | **The GP's experiences with treating adolescents in general practice.** |
| **Managing the knee pain.**<br>- Accepting KP.<br>- Putting KP into words.<br>- Decision-making.<br>- Finding the limit & maintaining balance.<br>- Getting a name.<br>- Searching for information.<br>- Desire for a solution (treatments)<br>- Exercises.<br>- Pain medicine. | **The parental role (main).**<br>**The parents' challenges.**<br>- Understanding of knee pain.<br>- Parents' knowledge of pain.<br>**The parents' responsibilities.**<br>- Parents as advocates.<br>- Parents as supporters.<br>- Parents as whips.<br>- Parents as interpreters of adolescents' pain. | **The GP's challenges (main).**<br>**The treatment situation.**<br>- Reassurance.<br>- Finding and delivering treatments.<br>- Building decision-making competencies.<br>- Treatment alliances.<br>**Systemic/external challenges.**<br>- Time.<br>- Resources.<br>- Understanding patients' history.<br>- Following up on treatment. |
| **Managing the social impact of knee pain.**<br>- The downwards spiral.<br>- Being limited.<br>- Being excluded.<br>- Economic aspects.<br>- Pain and symptoms.<br>- Stigma and mental work<br>- Not being taken seriously. | **The adolescents' perspective.**<br>- Social relations.<br>- Abilities and challenges.<br>- The adolescents wishes/dreams.<br>- The adolescents' feelings and experiences.<br>- Motivation. | **The challenges of the adolescents.**<br>- Worries.<br>- Exercises<br>- Articulating knee pain.<br>- Stigma/being misunderstood.<br>- Taking correct action on knee pain. |
| **Domains where KP emerges**.<br>- Sports and leisure activities.<br>- At home.<br>- School and education.<br>- Other situations.<br><br>**Thoughts on what made KP emerge.** | **Surveillance.**<br>**Parents as intermediaries.**<br>**Giving up control.** | **Domains where KP emerged.**<br>- The friend group.<br>- Sports and valued activities.<br>- General practice.<br>- Others. |
| **The roles of authorities.**<br>- Gym and sport coaches.<br>- Peers.<br>- Parents (parents as gatekeepers)<br>- Others.<br><br>**The historical aspects of Knee pain.** | **The parents experience of the extended treatment situation. (main)**<br>**The role of healthcare providers**.<br>- GP.<br>- Physiotherapist.<br>- The coach.<br>- Others.<br>**The GP-patient interaction.**<br>- Not being heard.<br>- Treatments. | **Different actors present in the treatment situation.**<br>- The parents' role.<br>- The role of the physiotherapist.<br>- The GP's responsibilities.<br>- Orthopaedic surgeon. |
| **The experience of the healthcare system.**<br>- Chiropractor.<br>- GP's (first experience & getting a name).<br>- Physiotherapists.<br>- Psychologist.<br>- Orthopaedic surgeon. | | **The consequences of treatments.**<br>- Reducing sports participation.<br>- Losing faith in treatments.<br>- Somatisation.<br>- Identifying serious pathologies. |
| **Visions for an mHealth app.**<br>- Visions for adolescents<br>- Visions for GPs<br>- Visions for surgeons<br>- Visions for PTs<br>- Visions for parents<br>- Visions for apps. | **Visions for an mHealth app.**<br>- Visions for adolescents.<br>- Visions for the GP<br>- Visions for parents<br>- Visions for the network | **Visions for an mHealth app.**<br>- Visions for GPs<br>- Visions for others<br>- Visions for parents.<br>- Visions for adolescents<br>- Visions for an app. |

**Appendix 7:** An overview of the main themes identified via the condensation and merger of themes and subthemes uncovered during the iterative coding of the datasets from the individual workshops. The condensation and merger identified 8 themes in the data from workshop 1, 1 main theme and 9 themes from workshop 2 and 1 main theme and 7 themes in the data from workshop 3. Reviewing the thematic structures revealed how adolescents, parents and GPs occupied different spaces within the treatment situation and how their challenges, roles and responsibilities differed.
