# Supplementary material for: Exploring User Visions for Modeling mHealth Apps Toward Supporting Patient-Parent-Clinician Collaboration and Shared Decision-making When Treating Adolescent Knee Pain in General Practice: Workshop Study
Source: JMIR Hum Factors. 2023 Apr 28;10:e44462. doi: 10.2196/44462 (PMC10182461; doi:10.2196/44462)
Supplement: Multimedia Appendix 7 [file humanfactors_v10i1e44462_app7.pdf]

Appendix 7 – Tension map.

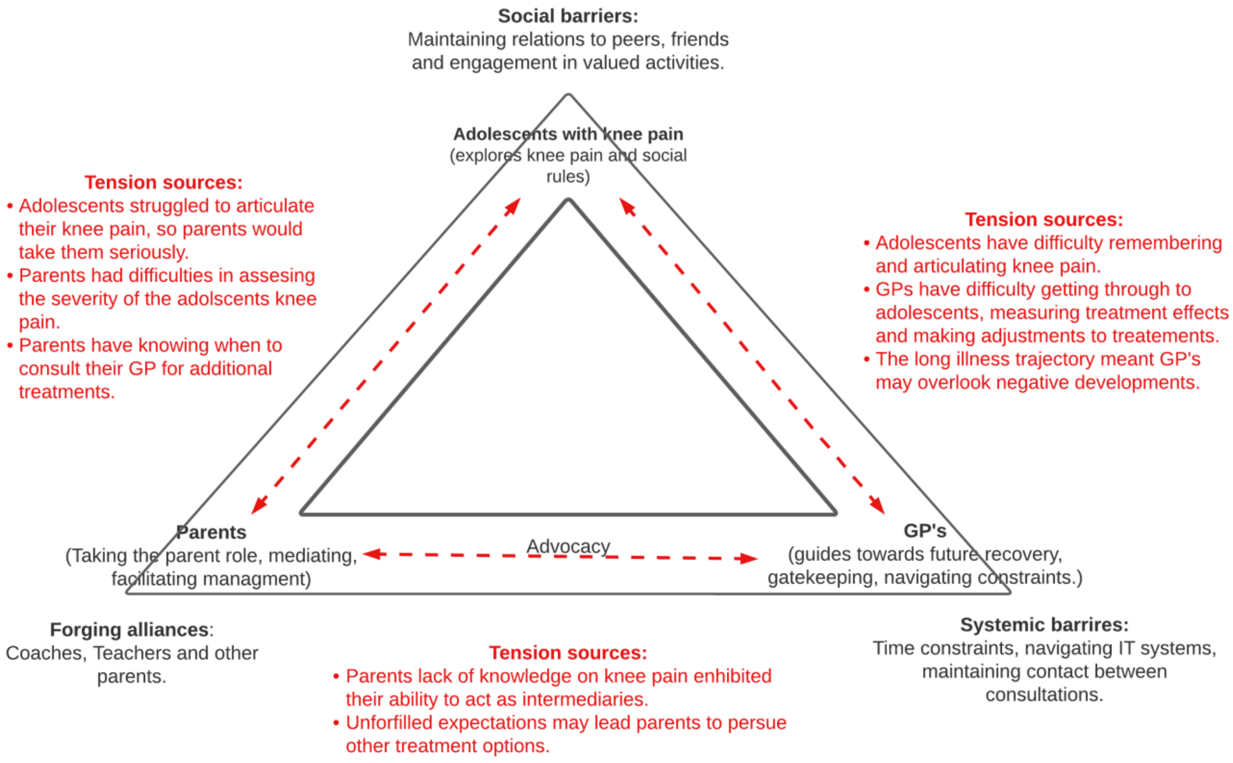

Appendix 8: The matrix analysis identified several tension sources in the collaborative space. By taking point in participants descriptions of their contextual challenges related to managing adolescents knee pain, several communicative barriers were identified, which caused tensions in the collaborative space and prompted withdrawal if left unaddressed. By taking point in the tensions as design opportunities, we drew on participants visions for an mHealth app to identify principles for an mHealth application for supporting shared decision-making.
