# Supplementary material for: Exploring User Visions for Modeling mHealth Apps Toward Supporting Patient-Parent-Clinician Collaboration and Shared Decision-making When Treating Adolescent Knee Pain in General Practice: Workshop Study
Source: JMIR Hum Factors. 2023 Apr 28;10:e44462. doi: 10.2196/44462 (PMC10182461; doi:10.2196/44462)
Supplement: Multimedia Appendix 8 [file humanfactors_v10i1e44462_app8.pdf]

Appendix 8 – Preliminary themes from plenary discussions.

	**Workshop 1:**<br>**Young adults**	**Workshop 2:**<br>**Parents**	**Workshop 3:**<br>**General Practitioners**
**Critique phase:**<br>**(challenges)**	- Knee pain creates a vicious circle.<br>- Finding the limit with knee pain.<br>- Learning to 'say it out loud'<br>- Accepting the knee pain.<br>- Securing parental support.<br>- Being taken serious by GP's.	- Adolescents struggle to articulate their knee pain.<br>- Knowing when to contact GP's.<br>- Providing exercise support.<br>- Knowing when to react to adolescents' knee pain.<br>- Discussing knee pain with GP.	-Adolescents struggle to remember and articulate knee pain.<br>- Losing contact with patients after consultations.<br>- No ability to adjust treatments.<br>-Adolescents forgets or misunderstands exercises.<br>- Time constraints.
**Fantasy phase:**<br>**(solutions)**	- Immediate help and support.<br>- Getting one diagnosis.<br>- One operation (not many)<br>- Psychological support.<br>- List of treatments (abroad)<br>- A physiotherapist in an app.<br>- Videos with exercises (short) and reminders.	- A feature for measuring pain developments.<br>- Motivation features.<br>- A preparation feature (before seeing the GP)<br>- A tool for informing the network.<br>- Patient information for parents.	- A feature, reminding adolescents to seek treatments.<br>- List of red flag symptoms.<br>- A journal feature for reflecting on developments of knee pain.
**Implementation phase:**<br>**(visions)**	- App with a profile.<br>- A scale for tracking knee pain.<br>- A Journal feature w. parent access.<br>- A feature (intranet) for communicating with GP and PT.<br>- A feature for tracking mood.<br>- Visualizations of knee pain developments.<br>- Adaptive suggestions for exercises.	- A to-do list for consulting the GP.<br>- App must be visible (web adds)<br>- Customization (motivating adolescents).<br>- GP or PT adjusted exercise program.<br>- Reminders for parents (exercises).<br>- Clearly defined goals for recovery.<br>- Chat rooms for knowledge sharing (Adolescents and parents).	- App should empower patient understanding of pain and maintaining treatment adherence.<br>- Three-legged design with information, exercises and self-tracking.<br>- A GP feature, with aggregated data for quick overview.

General Practitioners were abbreviated as GP's.
Physiotherapists were abbreviated as PT's.

Appendix 6: An overview of the themes (above) emerging during the workshops plenary discussions (below), distributed across the three phases of each individual workshops. The distribution of themes provided us with an indication on how the roles, tasks and challenges different within the treatment situation. Furthermore, the overview identified several overlaps and divergencies in the challenges experienced by adolescents, parents, and GP's during treatment situations. These insights were included to inform our analysis and synthesis of our data via the matrix.
